# First integrative trend analysis for a great ape species in Borneo

**DOI:** 10.1038/s41598-017-04435-9

**Published:** 2017-07-07

**Authors:** Truly Santika, Marc Ancrenaz, Kerrie A. Wilson, Stephanie Spehar, Nicola Abram, Graham L. Banes, Gail Campbell-Smith, Lisa Curran, Laura d’Arcy, Roberto A. Delgado, Andi Erman, Benoit Goossens, Herlina Hartanto, Max Houghton, Simon J. Husson, Hjalmar S. Kühl, Isabelle Lackman, Ashley Leiman, Karmele Llano Sanchez, Niel Makinuddin, Andrew J. Marshall, Ari Meididit, Kerrie Mengersen, Musnanda, Nardiyono, Anton Nurcahyo, Kisar Odom, Adventus Panda, Didik Prasetyo, Purnomo, Andjar Rafiastanto, Slamet Raharjo, Dessy Ratnasari, Anne E. Russon, Adi H. Santana, Eddy Santoso, Iman Sapari, Jamartin Sihite, Ahmat Suyoko, Albertus Tjiu, Sri Suci Utami-Atmoko, Carel P. van Schaik, Maria Voigt, Jessie Wells, Serge A. Wich, Erik P. Willems, Erik Meijaard

**Affiliations:** 1 The University of Queensland, School of Biological Sciences, Brisbane, QLD Australia; 2 ARC Centre of Excellence for Environmental Decisions, The University of Queensland, Brisbane, QLD Australia; 3 Borneo Futures, Bandar Seri Begawan, Brunei Darussalam; 4 Kinabatangan Orang-utan Conservation Programme, Sandakan, Sabah Malaysia; 5 Anthropology Program, University of Wisconsin Oshkosh, Oshkosh, WI USA; 6 Living Landscape Alliance, 5 Jupiter House Calleva Park, Berkshire, RG7 8NN United Kingdom; 7 School of Biological Sciences, University of Aberdeen, Zoology Building, Tillydrone Avenue, Aberdeen, AB24 2TZ United Kingdom; 8 CAS-MPG Partner Institute for Computational Biology, 320 Yue Yang Road, Shanghai, 200031 People’s Republic of China; 9 Max Planck Institute for Evolutionary Anthropology, Deutscher Platz 6, 04103 Leipzig, Germany; 10 Yayasan IAR Indonesia, Bogor, 16001 Indonesia; 11 Department of Anthropology, Stanford University, Stanford, California, USA; 12 Orangutan Tropical Peatland Project, The Center for International Cooperation in the Sustainable Management of Tropical Peatlands (CIMTROP), University of Palangka Raya, Central Kalimantan, Indonesia; 13 Departments of Anthropology and Biological Sciences, Program in Integrative and Evolutionary Biology (IEB), University of Southern California, Los Angeles, USA; 14 GFA/KWF, Kapuas Hulu Program, West Kalimantan, Indonesia; 15 Organisms and Environment Division, Cardiff School of Biosciences, Cardiff University, Cardiff, United Kingdom; 16 Danau Girang Field Centre, c/o Sabah Wildlife Department, Wisma Muis, 88100 Kota Kinabalu, Sabah Malaysia; 17 The Nature Conservancy (TNC) Indonesia, Jakarta, Indonesia; 18 Research Centre in Evolutionary Anthropology, and Palaeoecology, School of Natural Sciences and Psychology, Liverpool John Moores University, Byrom Street, Liverpool, L3 3AF United Kingdom; 19 German Centre for Integrative Biodiversity Research (iDiv), Halle-Jena-Leipzig, Germany; 20 Orangutan Foundation, London, United Kingdom; 21 Department of Anthropology, Program in the Environment, and School for Natural Resources and Environment, University of Michigan, Ann Arbor, MI 48109 USA; 22 Biology Faculty, Universitas Nasional (UNAS), Jakarta, Indonesia; 23 World Wide Fund for Nature-Indonesia (WWF-Indonesia), Central Kalimantan Program, Indonesia; 24 Science and Engineering Faculty, Queensland University of Technology, Brisbane, QLD Australia; 25 Austindo Nusantara Jaya Tbk, Jakarta 12910, Indonesia; 26 College of Arts and Social Sciences, The Australian National University, Canberra, ACT Australia; 27 Borneo Orangutan Survival Foundation (BOSF), Nyaru Menteng, Central Kalimantan, Indonesia; 28 The Indonesian Association of Primatologists (PERHAPPI), Bogor, Indonesia; 29 Flora and Fauna International-Indonesia, Ragunan, Jakarta Indonesia; 30 Faculty of Veterinary Medicine, Gadjah Mada University (UGM), Yogyakarta, 55281 Indonesia; 31 Lembaga Living Landscapes Indonesia (LLI), Pontianak, West Kalimantan Indonesia; 32 Psychology Department, Glendon College of York University, 2275 Bayview Avenue, Toronto, M4N 3M6 ON Canada; 33 Yayasan Orangutan Indonesia (YAYORIN), Pangkalan Bun, Central Kalimantan Indonesia; 34 Restorasi Habitat Orangutan Indonesia (RHOI), Bogor, West Java Indonesia; 35 World Wide Fund for Nature-Indonesia (WWF-Indonesia), West Kalimantan Program, Indonesia; 36 Forum Orangutan Indonesia (FORINA), Bogor, West Java Indonesia; 37 Anthropological Institute and Museum, University of Zurich, Zurich Switzerland; 38 Institute for Biodiversity and Ecosystem Dynamics, University of Amsterdam, Sciencepark 904, Amsterdam, 1098 Netherlands

## Abstract

For many threatened species the rate and drivers of population decline are difficult to assess accurately: species’ surveys are typically restricted to small geographic areas, are conducted over short time periods, and employ a wide range of survey protocols. We addressed methodological challenges for assessing change in the abundance of an endangered species. We applied novel methods for integrating field and interview survey data for the critically endangered Bornean orangutan (*Pongo pygmaeus*), allowing a deeper understanding of the species’ persistence through time. Our analysis revealed that Bornean orangutan populations have declined at a rate of 25% over the last 10 years. Survival rates of the species are lowest in areas with intermediate rainfall, where complex interrelations between soil fertility, agricultural productivity, and human settlement patterns influence persistence. These areas also have highest threats from human-wildlife conflict. Survival rates are further positively associated with forest extent, but are lower in areas where surrounding forest has been recently converted to industrial agriculture. Our study highlights the urgency of determining specific management interventions needed in different locations to counter the trend of decline and its associated drivers.

## Introduction

The Bornean orangutan (*Pongo pygmaeus*) is one of only two great ape species found in Asia today. The species is protected under both Malaysian and Indonesian law and is currently classified as Critically Endangered according to the IUCN Red List^1^. Despite strong public and scientific interest in orangutans in addition to considerable efforts and spending to conserve the species, we do not have an accurate assessment of the rate of Bornean orangutan population decline, or the drivers of this decline. Over the years, different estimates of population sizes have been proposed by various authors (Table 1), leading to confusion about the conservation status of the species. As for many threatened species, the rate of decline and the drivers of population change of orangutans are difficult to assess because of the species’ cryptic behavior, and also because surveys of orangutans are typically restricted to small geographic areas, are conducted over short time periods and employ different survey protocols.

**Table 1:** Total population estimates of the Bornean orangutan (*Pongo pygmaeus*) made by various authors.

Time period	Population range estimates	Authors
1961–1970	1,000–4,000	Harrisson^71^, Schaller^72^ and Reynolds^73^
1971–1980	15,000–90,000	Rijksen^74^
1981–1990	37,000–156,000	MacKinnon^75^
1991–2000	19,000–65,000	Rijksen & Meijaard^11^, MacKinnon^76^ Sugardjito & van Schaik^77^
2001–2010	54,000–62,675	Wich *et al*.^13^ and Singleton *et al*.^78^
2011–2015	>100,000	Wich *et al*.^18^

Extensive parts of the orangutan range in Borneo are remote and difficult to survey^2^. Orangutan abundance is often estimated from nest count surveys^3^, and a diverse range of survey protocols are employed for this purpose. Ground transect surveys of orangutan nests are the most commonly employed method^4–7^, but aerial surveys of orangutan nests using a helicopter have also been successfully used in Sabah to document the exact range and population size of the species throughout the state^6, 8, 9^. Surveys of orangutan nests are nevertheless typically restricted to accessible areas and often target locations with prior knowledge of orangutan occurrences, influencing the accuracy of population size estimates derived from nest count surveys^6^.

Interview surveys have also been used to assess orangutan occupancy^10, 11^. Because interview surveys are considerably cheaper to conduct than nest count surveys, they can cover considerably larger areas, even in locations without prior orangutan occurrence reports. For instance, a recent interview survey of orangutan sightings conducted by Meijaard *et al*.^10^ was able to cover 540 villages across the provinces of Kalimantan (Indonesian Borneo) and the Malaysian state of Sabah, with ten adult respondents sampled from each village. Despite its promise, this approach is subject to an array of biases associated with respondent data^10^. For example, in a forest where orangutans truly exist, the chance of orangutan sightings being reported by a respondent of a village near the forest will likely depend on the frequency of the respondent entering the forest. Accounting for variables that may influence the detection probability from each respondent can potentially minimize the bias in orangutan occupancy rate estimations from interview surveys. Furthermore, combining interview surveys of orangutan sightings and field surveys of orangutan nests can potentially provide a robust measure of the population changes through time, but this approach has never been applied to orangutans or to other ape species.

Density estimates based on orangutan nest counts are generally estimated via the Distance sampling method^12^ (e.g. refs 5, 7–9, 13–15). An alternative approach is to link nest density estimates or occurrence data to a suite of environmental predictors via static species distribution modeling techniques^16, 17^ (e.g. refs 18–20). Extrapolating spatial and temporal projections of orangutan density to unsurveyed locations is complicated, however, by the variable nature of nest construction and decay^21^. Nest decay rates have been shown to vary spatially depending on forest type and altitude^22^ and the rate of nest production is determined by the level of forest disturbance, e.g. by logging^23^. Caution is therefore required when projecting future orangutan distribution or abundance using standard species distribution modeling approaches based on nest count data, as the conclusions are potentially misleading.

Lowland natural forests (i.e. primary old-growth forest and degraded forests that have not been clear cut) with an altitude <500 m above sea level have been identified as the primary habitats for orangutans on Borneo^13, 24^. This is primarily because the composition and structure of lowland forests supports the productivity of wild tropical fruits, which are an important component of the diet of this species. The amount of rainfall during dry and wet seasons plays an important role in determining the phenology of fruiting trees important for orangutans^25^. A recent study by Wich *et al*.^18^ further restricted the orangutan range to lowlands outside the area with high mean annual rainfall, as high rainfall leaches soils which leads to less productive forests. Rainfall is also an important determinant of agricultural productivity and thus rural livelihoods on Borneo^26^, with optimal productivity occurring in areas receiving 7–9 consecutive wet months (>200 mm per month) and 2–3 consecutive dry months (<100 mm per month)^27^. Despite its apparent importance, however, seasonal rainfall patterns have rarely been taken explicitly into account in determining the extent of orangutan populations (but see ref. 19).

Contemporary anthropogenic factors have accelerated the decline of orangutans over the last centuries^28, 29^, with threats including habitat loss and fragmentation due to conversion of forest to other types of land use (such as agriculture, mining and infrastructure development), killing as a result of human-orangutan conflict, and hunting for bushmeat and wildlife trade (by killing females and capturing infants)^18, 30–33^. Forest loss has been primarily driven by conversion to agricultural plantations that occurred within the boundaries of industrial plantation concessions, but not so much by logging activities within the boundary of logging concessions on natural forest^34, 35^. Recent studies from Kalimantan suggest that human-orangutan conflict and its related killings increase with proximity to newly converted forest to industrial agriculture^31–33^. The tendency of village communities to hunt orangutans for bushmeat was found to be driven by complex socio-economic circumstances. Hunting tends to increase with a decrease in forest cover surrounding the village and an increase in area for agriculture in the village but a decrease in income from this sector^32, 33^. The proportion of Muslim populations was also found to represent a religious constraint on orangutan hunting for meat consumption^32, 36^.

Because of the challenges associated with surveying and modelling the population trends and drivers of population change of Bornean orangutans (or other species), we developed a dynamic abundance modelling methodology. Our integrated dynamic population model was applied within a hierarchical Bayesian framework^37^ and can (a) project the density of orangutans based on nest counts, (b) simultaneously integrate multiple types of data (i.e. nest counts from ground and aerial line transect surveys, presence-absence data from line transects and targeted surveys, and observations from interview surveys), and (c) explicitly account for the detection error inherent in each survey methodology due to associated effort. Using this novel approach we assessed the abundance and distribution of the Bornean orangutan through time and determined the contribution of climate and land use dynamics to the changes observed.

## Results

### Model diagnostics and performances

Prior to fitting the model to the data, we tested for correlation among the original (unstandardized) variables and among the standardized environmental variables explaining the initial abundance, occupancy and survival rates, and found weak correlations among these variables (with absolute Pearson correlation <0.45, see Supplementary Table 1). The WinBUGS simulation converged well, as confirmed by the value of Rhat (ranged between 1 and 1.1) for all parameters, and the absence of seasonality within each Markov chain Monte Carlo (MCMC) chain plot and overlap between the three chains (Supplementary Figure 1). We also detected no apparent correlations between the posterior distributions of the coefficients of the linear and the quadratic terms for altitude (*ALT*), the longterm mean monthly rainfall during the dry season (*DRY*) and wet season (*WET*) (Supplementary Figure 2), which suggests the reliability of the estimated coefficients obtained for these variables.

Our dynamic abundance model performed well with a good correspondence between the simulated nest predictions and the actual observations. The average Pearson correlation coefficient for all time periods is *r* = 0.828 (with *r*_1997–2002_ = 0.824, *r*_2003–2008_ = 0.818 and *r*_2009–2015_ = 0.841) and the average *R* ^2^ is 0.804. The model also has a good correspondence between the simulated orangutan presence-absence and the actual observation obtained from interview surveys, with Sensitivity SN = 0.812 and Specifity SP = 0.726.

### Survey specific parameters

The probability of detecting orangutan nests from field surveys per km^2^ varied depending on respective survey protocol (Table 2). Aerial transects surveys had the highest probability of detecting orangutan nests (logit(1.516)^−1^ = 82%), followed by the ground transect surveys (75%). This could be because aerial surveys were usually conducted in areas with prior knowledge of orangutan occurrences due to the cost of operating the helicopter. The occurrence data of the combined aerial and ground line transects and other targeted surveys had a lower probability of detecting the nests (64%).

**Table 2:** Posterior means and the 95% credible interval (CI) of the mean for each parameter explaining the latent orangutan population density (first level of the orangutan dynamic abundance model), the observed orangutan occurrence (second level of the orangutan dynamic abundance model), latent orangutan nest density (third level), and the observed orangutan nest density and occurrence (fourth level).

Model level and sub-model	Scale (Prior)	Variable (Parameter)	Posterior parameter	
			Mean	95% CI
***First level: Latent orangutan population density***				
Initial abundance in 1997–2002 (*λ*_*i*_ in Eq. (1))	Log (U[−8,8])	Intercept (*α*_1_)	1.023	(0.901, 1.151)
		*ALT* (*α*_2_)	0.021	(0.001, 0.047)
		*ALT* ^2^ (*α*_3_)	−0.025	(−0.051, −0.004)
		*DRY* (*α*_4_)	3.781	(3.162, 4.331)
		*DRY* ^2^ (*α*_5_)	−3.892	(−4.102, −3.662)
		*WET* (*α*_6_)	3.951	(2.920, 4.614)
		*WET* ^2^ (*α*_7_)	−4.162	(−4.621, −3.712)
		*DPA*_1_ (*α*_8_)	−0.072	(−0.114, −0.024)
		*MS* (*α*_9_)	0.001	(0.000, 0.004)
		*FR*_1_ (*α*_10_)	0.881	(0.621, 1.161)
		*FR*_1_ × *CFA*_1_ (*α*_11_)	0.071	(0.022, 0.112)
Occupancy rates (*φ*_*i*,*t*_ in Eq. (2))	Logit (U[−6,6])	Intercept (*β*_1_)	1.423	(1.361, 1.489)
		*ALT* (*β*_2_)	0.181	(0.085, 0.271)
		*ALT* ^2^ (*β*_3_)	−0.123	(−0.227, −0.023)
		*DRY* (*β*_4_)	3.621	(3.243, 3.991)
		*DRY* ^2^ (*β*_5_)	−3.422	(−3.842, −3.012)
		*WET* (*β*_6_)	3.049	(2.641, 3.449)
		*WET* ^2^ (*β*_7_)	−3.664	(−4.021, −3.304)
		*DPA*_*t*_ (*β*_8_)	−0.036	(−0.093, 0.014)
		*MS* (*β*_9_)	0.005	(0.001, 0.006)
		*FR*_*t*_ (*β*_10_)	0.872	(0.511, 1.236)
		*FR*_*t*_ × *CFA*_*t*_ (*β*_11_)	0.049	(0.014, 0.079)
***First level: Latent orangutan population density***				
Survival rates (*θ*_*i*,*t*_ in Eq. (3))	Logit (U[−4,4])	Intercept (*η*_1_)	2.662	(2.412, 2.902)
		*ALT* (*η*_2_)	−0.017	(−0.052, 0.015)
		*ALT* ^2^ (*η*_3_)	0.025	(0.005, 0.053)
		*DRY* (*η*_4_)	−0.788	(−1.315, −0.248)
		*DRY* ^2^ (*η*_5_)	0.721	(0.146, 1.301)
		*WET* (*η*_6_)	−0.514	(−1.164, 0.116)
		*WET* ^2^ (*η*_7_)	0.537	(0.017, 1.047)
		*DPA*_*t*_ (*η*_8_)	−0.136	(−0.161, −0.110)
		*MS* (*η*_9_)	0.012	(0.000, 0.026)
		*FR*_*t*_ (*η*_10_)	0.133	(0.052, 0.212)
		*FR*_*t*_ × *CFA*_*t*_ (*η*_11_)	0.215	(0.101, 0.324)
Recruitment rate (*δ*_*i*,*t*_ in Eq. (4))	Log (U[−6,6])	Intercept (*χ*)	−2.265	(−2.317, −2.215)
***Second level: Observed orangutan occurrence***				
Orangutan detection rate from interview surveys (*ρou*_*i*,*m*,*t*_ in Eq. (7))	Logit	Intercept (*υ*_1_)	−1.726	(−1.982, −1.476)
	(U[−4,4])	*FE*_*m*_ (*υ*_2_)	0.417	(0.197, 0.647)
***Third level: Latent orangutan nest density***				
Scaling factor of nest counts and orangutan density (*ψ*_*i*,*t*_ in Eq. (6))	Normal (U[−10,10])	Intercept (*γ*_0_)	2.279	(2.092, 2.459)
		*MGV* (*γ*_1_)	0.385	(0.041, 0.725)
		*PT* (*γ*_2_)	−0.193	(−0.302, −0.093)
		*LOWL* (*γ*_3_)	0.165	(−0.036, 0.369)
		*MONT* (*γ*_4_)	0.079	(−0.102, 0.264)
		*FRGM* (*γ*_5_)	−0.153	(−0.251, −0.063)
***Fourth level: Observed orangutan nest density and occurrence***				
Nest detection rate from line transect surveys (density) (*ξ*_*i*,*j*,*t*_ in Eq. (8))	Logit	Intercept (*μ*_aerial_)	1.516	(1.115, 1.920)
	(U[−4,4])	Intercept (*μ*_ground_)	1.097	(0.715, 1.481)
Nest detection rate from line transect and targeted surveys (occurrence) (*ρnest*_*i*,*k*,*t*_ in Eq. (9))	Logit (U[−4,4])	Intercept (*ζ*)	0.574	(0.198, 0.944)

The probability of detecting orangutans via interview surveys was 15% on average if the respondent entered the forest less than once per month and 21% if they entered the forest more frequently (Table 2). The reason for low detection rates of orangutans from interview survey, in comparison to the nests from field survey, is twofold: (1) orangutans are much less common than their nests, and (2) nest count surveys are generally targeted at areas with prior knowledge of orangutan occurrences due to cost constraints.

Nest decay rate was estimated to be 228 days on average for Borneo (Table 2). This however varied slightly across different forest types, where mangrove forest had the longest time to decay (266 days), followed by lowland forest (244 days), montane forest (236 days), and peat forest (209 days).

### Orangutan abundance by region and land use

**Figure 1:**
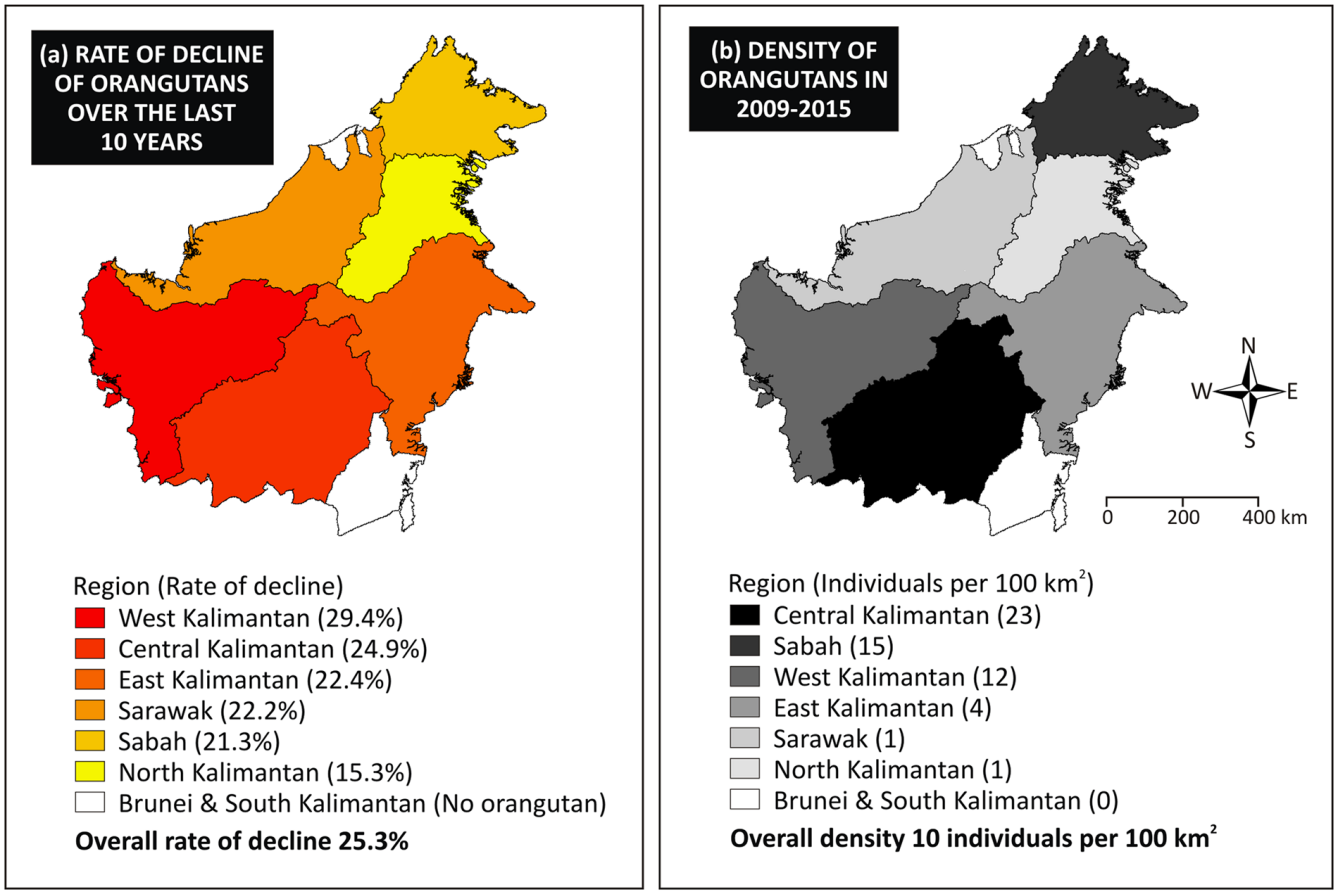
Rate of decline of the Bornean orangutan over the last ten years (**a**) and the estimates of orangutan density by region (**b**). These maps are available at https://figshare.com/s/c8ec56a72628f256b3a8.

The dynamic abundance model estimated that the density of Bornean orangutans has declined by 25% over the last ten years (Fig. 1a). We estimated the overall density of orangutans over Borneo in the period 1997–2002 was about 15 individuals per 100 km^2^, but the density was reduced to 10 individuals per 100 km^2^ in 2009–2015 (Supplementary Table 2). We estimated that Central Kalimantan had the highest density of orangutans during 1997–2015, followed by Sabah, West Kalimantan, East Kalimantan, Sarawak, and North Kalimantan (Fig. 1b and Supplementary Table 2).

**Figure 2:**
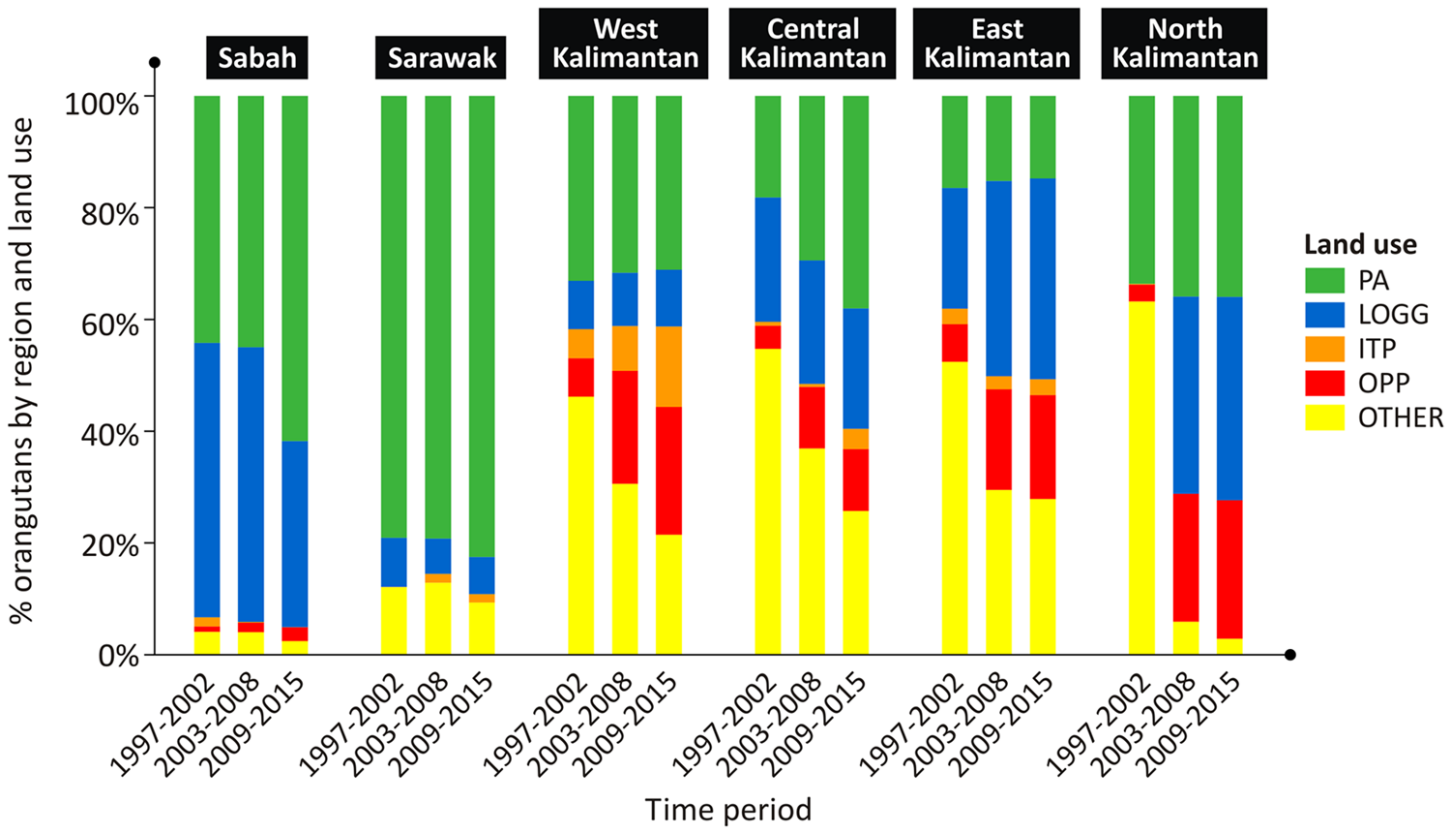
Distributions of orangutan populations across different regions and land uses in three consecutive time periods between 1997 and 2015. Land use appraised include protected areas (PA), logging concessions on natural forest (LOGG), industrial timber plantation concessions (ITP), oil palm concessions (OPP), and outside protected areas, infrastructure and urban areas and without concession (OTHER).

The distribution of orangutan populations across different land uses varied across regions. In Sabah and Sarawak, most of the orangutan populations resided within the boundaries of protected areas (PA) and logging concessions on natural forests (LOGG) (Fig. 2). In Kalimantan, the population generally resided within the boundaries of PA and LOGG and in areas without concessions (or classified as ‘OTHER’). Across the whole of Borneo, the proportion of orangutans residing within the boundary of PA has increased through time (Fig. 2), mainly because the orangutan populations have gradually disappeared from other land uses and/or the extent of PA had increased recently^9, 10, 20^, e.g. with the establishment of the Sebangau National Park in Central Kalimantan, new contiguous protected forests between the Maliau Basin, Imbak Canyon and Danum Valley conservation areas in Sabah, and several new protected areas around the BALE (Batang Ai National Park and Lanjak Entimau Wildlife Reserve) landscapes in Sarawak.

### Drivers of changes in orangutan abundance

**Figure 3:**
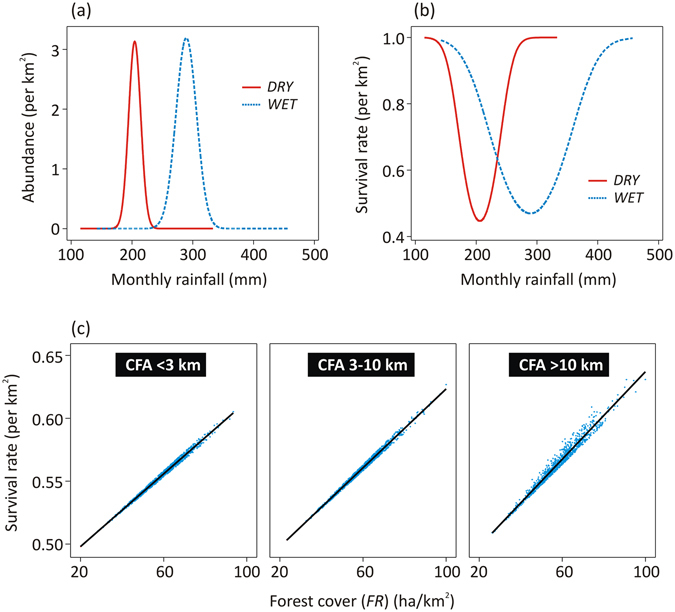
The effect of seasonal rainfall, forest cover, and distance to forest recently converted to industrial agriculture, on the orangutan abundance and survival rates. The relationship between the monthly mean rainfall during the dry season (*DRY*) and wet season (*WET*) on the orangutan abundance in the initial time period 1997–2002 (**a**) and the survival rate every six years between 1997 and 2015 (**b**). The effect of forest cover (*FR*) on orangutan survival rate, with varying distances to forest recently converted to industrial agriculture (*CFA*) (**c**).

The initial abundance of orangutans per km^2^ was most strongly associated with the amount of rainfall during both wet (*WET*) and dry seasons (*DRY*), with the greatest abundance observed in areas of intermediate rainfall during each season (Table 2 and Fig. 3a). Survival rates also correlated most strongly with the amount of rainfall during both wet (*WET*) and dry seasons (*DRY*), however, with the rates being lowest in areas of intermediate seasonal rainfall (Table 2 and Fig. 3b). Natural forest extent (*FR*) was positively associated with the initial abundance and survival rates.

The interactions between natural forest extent and distance to forest recently converted to industrial agriculture (*FR* × *CFA*) was positively associated with survival rates, suggesting that survival rates are lowest in areas with fragmented forest and near to new areas of industrial agriculture, as the possibility of human-orangutan conflicts increase (Table 2 and Fig. 3c). Survival rates are also positively associated with proximity to protected areas (*DPA*), indicating that protected areas are mitigating some threats to orangutans (Table 2).

**Figure 4:**
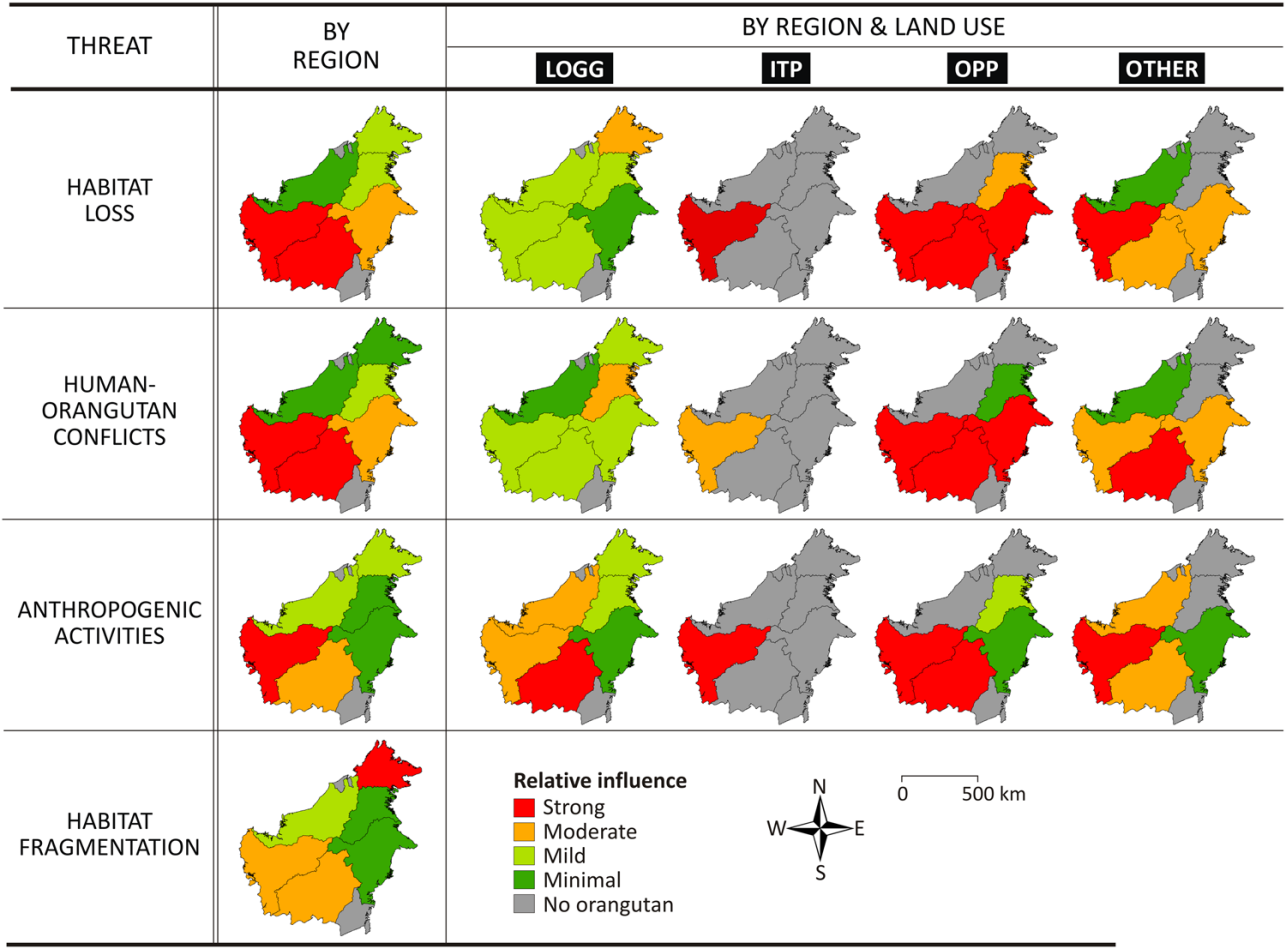
The relative importance of drivers of orangutan decline during 1997–2015 by region and land use. Drivers include habitat loss, human-orangutan conflicts, anthropogenic activities, and habitat fragmentation. Land uses appraised include logging concessions on natural forest (LOGG), industrial timber plantation concessions (ITP), oil palm concessions (OPP), and outside protected areas, infrastructure and urban areas and without concession (OTHER). Level of importance was assessed based on percentile values of the associated threat across different regions (**a**), and across different regions and land uses (**b**), i.e. Strong (red): >75th percentile, Moderate (orange): 50–75th percentile, Mild (light﻿ green): 25–50th percentile, and Minimal (dark green): <25th percentile.

Based on variables explaining survival rates, we assessed drivers of orangutan population decline during 1997–2015 in each region, and this includes habitat loss, human-orangutan conflicts, anthropogenic activities, and habitat fragmentation (Fig. 4). For Sabah, we estimated that orangutan population decline is driven by (1) moderate rates of habitat loss within the boundaries of LOGG, and (2) high levels of habitat fragmentation. For Sarawak, the decline is mainly driven by (1) moderate rates of habitat loss within the boundaries of LOGG, and (2) moderate anthropogenic pressure within the boundaries of LOGG and OTHER. For East and North Kalimantan, orangutan population declines were mainly driven by (1) moderate to high rates of habitat loss and (2) moderate to high intensities of human-orangutan conflicts within the boundaries of oil palm plantation concessions (OPP) and OTHER. For West and Central Kalimantan, drivers of decline include (1) moderate to high rates of habitat loss and (2) moderate to high intensities of human-orangutan conflicts within the boundaries of industrial timber plantation concessions (ITP), OPP and OTHER, (3) moderate to high anthropogenic pressure within the boundaries of ITP, OPP, LOGG and OTHER, and (4) moderate levels of habitat fragmentation.

## Discussion

Our analysis is the first robust population trend analysis for orangutans or other great ape species that includes quantitative assessments of drivers of change. Methodological challenges associated with determining spatial and temporal variation in ape density across large areas have so far made such studies infeasible, but our novel approach has overcome these challenges. Our analysis advances current estimates by providing the underlying population trend through time, with the species estimated to have declined at an alarming rate of 25% over the past 10 years. This contradicts crude population estimates proposed by different authors that have indicated an increasing number of orangutans across the island, reflecting increasingly available data on the species and associated survey efforts and not an absolute increase of orangutans (Supplementary Figure 3). This is mainly because the previous studies were conducted separately for each time period and they failed to take into account the dynamic process affecting the orangutan population change.

### Orangutan abundance and competition from humans in area with intermediate rainfall

Our model indicates that the long-term abundance of orangutans per km^2^ is strongly determined by seasonal rainfall, with the species being most abundant in areas receiving intermediate rainfall during the dry season (150–250 mm per month from May to September) (Fig. 3a) and the wet season (200–400 mm per month from November to March). This is comparable to Indonesian agro-climatic zone B with 7–9 consecutive wet months (>200 mm per month) and around two consecutive dry months (<200 mm per month)^27^. This area essentially receives the right amount of rain throughout the year and is likely able to support plenty of wild tropical fruits essential for orangutans, such as *Moraceae* (figs) and *Anacardiaceae* (mangos)^38, 39^. The extent of the intermediate rainfall zone on Borneo is smaller than the extent of lowlands with altitude <500 m above sea level (Supplementary Figure 4), the range that has long been recognized as the primary niche for Bornean orangutans^11^. The extent is also smaller than the area of low-moderate mean annual rainfall (Supplementary Figure 4) recently suggested by Wich *et al*.^18^. For example, most lowlands in Sarawak are outside the intermediate rainfall zone, as are the lowlands in the western region of Sabah and in the east of East Kalimantan (Supplementary Figure 4). Although orangutan populations may be found in some of these areas, their densities are low. The zone of intermediate rainfall mainly occurs in Central and West Kalimantan, the two provinces with currently the largest orangutan populations outside protected areas. In Sarawak, the zone of intermediate rainfall also occurs around the Batang Ai National Park and Lanjak Entimau Wildlife Reserve, where most of the orangutan populations in this state currently reside.

Besides being important for orangutans, areas with intermediate rainfall are also important for people. The climate in this zone optimally supports plant productivity and agriculture, allowing year-round cultivation of crops, fruits and vegetables^27^. This is supported by the fact that the proportion of agricultural areas, i.e. plantations and agriculture fields and shrublands from abandoned agriculture, outside the government-sanctioned protected areas on Borneo, increases as they are located closer to zones with intermediate rainfall (Supplementary Figure 5d). Because orangutans and humans favor the same climate zone and range, orangutans are facing severe competition from humans, as confirmed by our model where the species survival rates were lowest in this zone (Fig. 3b). In this study we were able to include both altitude and rainfall seasonal pattern as predictors explaining abundance and survival rates because there are no strong correlations between these variables (Supplementary Table 1). Altitude (and its quadratic term) by itself was found to be a non-significant predictor, suggesting that altitude indirectly affects orangutan abundance and survival rates, most likely through rainfall.

While the relationship between rainfall and orangutan abundance is relatively easy to understand from the direct impact of intermediate rainfall on the abundance of wild fruits, the connections between rainfall and orangutan survival rates are more difficult to discern and are most likely related to multifaceted consequences of changing rainfall patterns as part of global climate change and anthropogenic land use change in this area, i.e. vast conversion of forest to agriculture^35, 40, 41^. Forest clearing has led to the loss of orangutan habitat, as well as the loss of livelihood for communities who greatly depend on forest goods. As climate becomes more erratic, periods of wild fruit scarcity may have increased and the intensity and frequency of forest fires (often originating in drained peat swamp areas) and flooding events (due to upstream deforestation) also increased^42, 43^. These severe environmental circumstances have most likely led to increased competition between humans and orangutans^20^. Displaced communities who cannot generate sufficient income from agriculture may seek other income opportunities such as hunting and poaching, or are more sensitive to conflicts with orangutans over crop-raiding^44^.

The link between areas with intermediate rainfall and hunting propensity can be explained in light of recent research, suggesting that hunting tends to increase with a decrease in forest cover surrounding settlements and an increase in area for agriculture around settlements but a decrease in income from this sector^32, 33^. Based on population census and land cover data among administrative districts in Kalimantan, we found that districts located within the intermediate rainfall zone have the socio-economic features that lead to higher propensities of hunting compared to districts located outside these zones. The proportions of agricultural areas outside the government-sanctioned protected areas are generally higher in districts where large proportions of these areas overlap with intermediate rainfall range (Supplementary Figure 5a). As anticipated, the proportion of forest areas within the same zones is generally lower in these districts (Supplementary Figure 5b). As the proportions of agricultural areas overlapping with the intermediate rainfall zones in a district increases, the proportion of smallholder farmers decreases (Supplementary Figure 5c) but the proportion of workers engaged in agriculture activities increases (Supplementary Figure 5d). Despite being agriculturally rich, however, the percentage of people living in poverty is generally higher in these districts that derive lots of their income from industrial-scale agriculture (Supplementary Figure 5e). Also, the poverty-gap index is higher in these agriculturally rich districts (Supplementary Figure 5f), indicating that profits from agricultural development accrue to a small section of society. This indicates that the current orangutan hunting activities could be exacerbated by social and economic circumstances with displaced orangutans competing with small-holder farmers that have less and less land for their own agricultural activities. The connection between socio-economic background, particularly poverty, and hunting and poaching, is generally well known based on various case studies from Asia and Africa^39, 45^. However, the evidence for claims around poverty as a driver of hunting is weak, mainly because hunting has been overwhelmingly framed exclusively as an issue of conservation and biodiversity loss rather than of poverty and development^46^, but that does not mean that poverty is not an important factor.

Recent studies have also found that hunting tends to increase with a decrease of Muslim populations in the village, suggesting that religious affiliation potentially provides a barrier to current orangutan hunting^39, 42^. Based on census data, we found that agriculturally rich districts located within the intermediate rainfall range in Kalimantan generally have a large proportion of non-Muslim people (Supplementary Figure 5g). This is likely because the high agricultural value has long made these areas the primary home for large indigenous communities, most of which are non-Muslims. Thus, a low proportion of Muslim populations is likely confounded within an area’s high agricultural value, without necessarily influencing the propensity to hunting and orangutan survival rates. Furthermore, our model found a minimal impact of the percentage of Muslims within districts on orangutan survival, suggesting a weak correlation between religious affiliation per se and orangutan survival rates. Furthermore, earlier study suggests that hunting for bushmeat is not solely carried out by non-Muslims for their own consumptions, but also by various communities for selling the meat^39^, implying that the current hunting practices are also driven by economic incentives such as trade. To inform suitable strategies for abating orangutan hunting requires a better understanding of individual hunter motivations, and the anthropological and economic motives driving them^47^.

Increased contact with humans may also increase the risk of infectious disease in orangutans, which can affect the survival rates of the species in the wild. Previous serological studies suggest that exposure to human pathogens does occur both in free-ranging and semi-captive orangutans^48^. Pathogens, such as intestinal parasites, can be transmitted directly from humans^49^. In rehabilitation centers, overcrowding, abnormality in the population social structure, and dietary imbalances, can exacerbate disease transmission among orangutans^48^.

### Forest, conversion to industrial agriculture, and climate change

Our model indicates that the long-term abundance of orangutans and survival rates per km^2^ are strongly determined by the extent of natural forest. This suggests that the reduction of forest extent alone will decrease orangutan survival rates. The loss of natural forest was found to be an equally important driver of orangutan declines across all regions of Borneo during 1997–2015 (Fig. 4).

When threats from forest clearing are absent, such as in the case of populations within the boundary of protected areas, survival rates can also decline due to decreasing forest carrying capacity, e.g. increased period of wild fruit scarcity due to climate change. Both global climate change, and climatic changes directly driven by deforestation are predicted to impact rainfall patterns on Borneo, with some areas anticipated to experience significant rainfall reductions, such as prolonged consecutive dry months^50^. Isolated forest patches of orangutan habitats are particularly prone to extinction due to this type of disturbance. This is exactly the issue currently faced by orangutan populations in Sabah. Comparison among orangutan habitat networks across different regions of Borneo shows that the average size of forest patches where orangutans currently reside are lowest for Sabah (Supplementary Figure 6a) and the distance between forest patches is also largest for this region (Supplementary Figure 6b), suggesting that the populations in this state face the highest risks due to habitat fragmentation (Fig. 4). Hence, although large proportions of orangutan populations in Sabah currently reside within the boundary of PAs, threats from global climate change and other disturbance such as disease, as described earlier, can potentially annihilate orangutan populations within a PA due to relatively small PA size and lack of connectivity among orangutans’ habitats within the current PA networks^51, 52^.

Our model also found that survival rates were determined by the interaction between forest extent and proximity to forest recently converted to industrial agriculture. This is likely to be directly related to the increased possibility of human-orangutan conflicts, such as crop-raiding, over newly established large-scale industrial agriculture and hence killing of crop-raiding individuals^53^. However, we also found that survival rates increase with proximity to PAs, indicating that forest protection is mitigating some threats to orangutans. Human-orangutan conflicts during 1997–2015 were found to be equally important drivers of orangutan declines across all regions of Borneo (Fig. 4). Although conflicts due to conversion of forest to industrial agriculture appear to occur most intensively in West and Central Kalimantan compared to other regions^31^, this is probably because large orangutan populations are found in these provinces, and thus does not necessarily imply that conflicts have a relatively minimal impact on populations in other regions.

Here, we addressed human-orangutan conflicts by assessing the interaction between forest cover and proximity to forest that has been recently converted to industrial agriculture. Conflicts become less frequent with time either because orangutans become less common or adapt to the new landscape^54^. This is what likely happened in extensive areas of lowland forests in Sabah that had high densities of orangutans prior to the 1960s when the forests were converted to oil palm. However, we did not take into account the possibility that the frequency of conflicts may also vary depending on fruit scarcity. As rainfall is predicted to be more extreme in the future, increased periods of wild fruit shortages are anticipated and this could potentially affect orangutan crop-raiding behavior.

## Conclusion

Orangutan populations on Borneo have declined at a rate of 25% over the last 10 years. Pressure on orangutan populations in the same period of time varied substantially among regions, with the populations in Sabah, Sarawak, East and North Kalimantan experiencing relatively moderate pressure, as opposed to high pressure in West and Central Kalimantan. The co-occurrence of orangutan populations with areas most suitable for human activities has led to an enhanced risk of human-wildlife conflicts. Unless threats from climate change, land use change and other anthropogenic pressure are abated, we predict that most populations of the Bornean orangutan will be severely impacted by human activities.

Poor connectivity among orangutan habitats between the boundaries of PAs is currently the predominant threat to orangutan populations in Sabah. Orangutan populations in Sarawak, East and North Kalimantan face the same threats as West and Central Kalimantan due to habitat loss from continuing forest conversion to industrial agriculture and human-orangutan conflicts, but the latter two areas also suffer additionally from anthropogenic activities.

As the populations in different regions face different threats, specific abatement plans should be implemented to ensure the long-term persistence of the species. This includes (1) maintaining high forest cover in orangutan habitats and improving the connectivity among the remaining forest patches where orangutans live through better spatial planning for all regions of Borneo, (2) close cooperation with plantation companies, smallholder farmers and wider communities in managing conflicts with orangutans in Kalimantan, and specifically in West and Central Kalimantan (3) improving the effectiveness of anti-hunting efforts and education and (4) developing a better understanding of the underlying socio-economic motivations of hunting.

## Methods

### Study area

**Figure 5:**
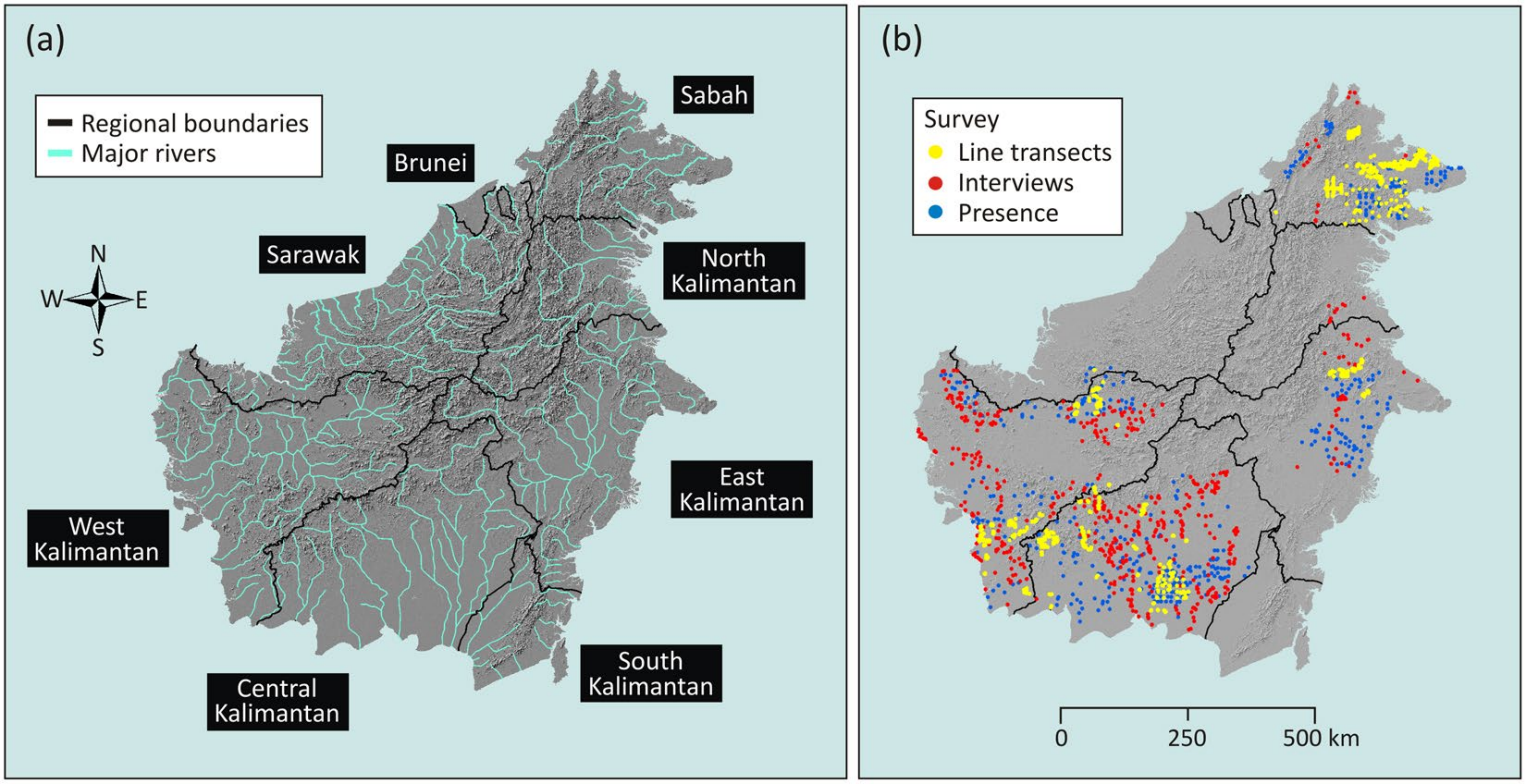
Maps of the study area and orangutan surveys. A topographic map of Borneo with regional boundaries and rivers, derived from the SRTM digital elevation data^67^ district maps provided by the Indonesian Geospatial Information Agency^68^ and GADM database of Global Administrative Areas^69^ and river networks provided by the HydroSHEDS^70^ and visual inspection via Google Earth (**a**). The locations of orangutan surveys conducted over the last two decades: line transect surveys of orangutan nests (ground and aerial), interview surveys of direct orangutan sightings, and presence points of nest and individual sightings (**b**). These maps are available at https://figshare.com/s/4ca9f2ae131d6a201751.

Borneo is the third-largest island in the world (approximately 740,000 km^2^) and is shared by the Malaysian states of Sabah and Sarawak and the sultanate nation of Brunei in the north, and by Indonesian provinces in the south (i.e. West, Central, South and East Kalimantan; the latter was recently divided to establish North Kalimantan province) (Fig. 5a). The island is largely mountainous, with mountains branching westward from the central core along the border between Sarawak and West Kalimantan, and a discontinuous series of mountain ranges running parallel to the east and southeast coasts of the island) (Fig. 5a). Borneo’s interior is largely mountainous but extensive lowlands and swamps occur along the coasts. A large part of Borneo is drained by navigable rivers, which represent the principal and sometimes only routes for trade and commerce, but also present barriers to orangutan dispersal^55, 56^. The main rivers are the Kapuas in West Kalimantan, the Barito and Kahayan in Central Kalimantan, the Mahakam and Kayan in East Kalimantan, the Rajang and Baram in Sarawak, and the Kinabatangan in Sabah.

We divided Borneo into grid cells with a spatial resolution of 1 × 1 km^2^, and excluded Brunei and South Kalimantan as they are outside the known orangutan range. This resolution allows us to simulate orangutan dispersal from each focal cell (100 ha) to eight neighboring grid cells, resulting in a 3 × 3 km^2^ dispersal block (900 ha). This resolution conforms roughly to the home ranges of female Bornean orangutans, which vary between 150 and 850 ha^57^.

### Orangutan data

We utilized two types of orangutan data: nest counts and presence-absence data. The nest count data were obtained from line transect surveys (aerial and ground) (Fig. 5b). The presence-absence data were derived from two survey approaches: (1) line transect (aerial and ground) and targeted surveys of nest observations, and (2) interview surveys of direct orangutan sightings (Fig. 5b). For each survey method, we divided the data into three time periods: (1) 1997–2002, (2) 2003–2008, and (3) 2009–2015, thus providing an analysis of the change in orangutan abundance every six years. This time interval conforms to the minimum inter-birth intervals (the time between consecutive offspring) of female Bornean orangutans^58^. It also conforms roughly to the time frames of orangutan conservation plans at a national level for Indonesia^59^ and at state level for Malaysia^60^.

The aerial survey data mainly cover Sabah and were collected between 1999 and 2012 using helicopters following different flight routes, as described in Ancrenaz *et al*.^8, 9^, giving a total route length of approximately 2,200 km. The ground surveys were carried out sporadically between 1997 and 2015 across Borneo by various orangutan research teams and non-governmental organizations, giving a total transect length of approximately 1,200 km. The targeted surveys mainly include the reconnaissance walks, i.e. a walk following a predetermined direction through the survey area. These surveys followed a standard established methodology to detect and record the nests of great apes^3^.

To facilitate the use of nest count data collected from various methods of line transect surveys, we standardized the metric of orangutan nests to obtain a nest density estimate for each 1 × 1 km^2^ grid cell. For the ground surveys, we calculated the density of orangutan nests using the Distance sampling method, based on the perpendicular distance of each nest to the transect^12^. For the aerial surveys, the data were mainly in the form of an aerial index value (*AI*) describing the number of nests detected per km of flight. Following Ancrenaz *et al*.^8^, the density of orangutan nests per km^2^, i.e. *gnest*, can be estimated via: log(*gnest*) = 4.7297 + 0.9796 log(*AI*). Density estimates for each 1 × 1 km^2^ grid cell were then obtained by averaging the estimate across all aerial surveys conducted within the grid cell, giving approximately 6,500 of 1 × 1 km^2^ grid cells where orangutan nest surveys had been conducted across Borneo. These data were then used to form a matrix array of orangutan nest density *Y*_*i*,*j*,*t*_ comprising three matrices of survey period (*t*), with each matrix consisting of 6,500 rows of grid cells (*i*) and 2 columns of survey protocol (*j*), i.e. ground and aerial transects.

To derive the occupancy of nests in each 1 × 1 km^2^ grid cell from the ground and aerial transect and targeted surveys for each time period, we first divided the grid into sub-cells with the resolution of 200 × 200 m^2^. This is to avoid duplicated reports of the same clusters of nests. If at least one survey reported the occurrence of a nest within a sub-cell, we defined that orangutan nests were observed in this sub-cell. If no orangutan nests were recorded within the sub-cell in any of the surveys, we defined that orangutan nests were unobserved in this sub-cell. We then constructed a matrix array *Znest*_*i*,*k*,*t*_ comprising three matrices of survey period (*t*), and with each matrix comprising 6,500 rows of grid cells (*i*) and 25 columns of nest observations within sub-cells (*k*).

The interview surveys of orangutan sightings were conducted in 540 villages across Kalimantan and Sabah in 2008 and 2009, and verification surveys in 2011, with 10 respondents in each village, as described in Meijaard *et al*.^10^. Each respondent was asked how frequently he or she entered the forest around the village (i.e. more than once per month or less than once per month) and the last time they had seen an orangutan either in the forest or in the village (i.e. within this year or more than a year ago). Additionally, personal details of each respondent were recorded, including their age and how long they had resided in the village. Based on this information, we derived the occurrence (observed or unobserved) of orangutans in each 1 × 1 km^2^ grid cell and constructed a matrix array *Zou*_*i*,*m*,*t*_, comprising three matrices of survey period (*t*) with each matrix consisting of 540 rows of grid cells (*i*) and 10 columns of respondent observations (*m*). Because the chance of any respondent sighting an orangutan would likely depend on that respondent’s frequency of entering the forest, we also constructed a corresponding binary matrix *FE*_*i*,*m*_, coded as ‘1’ when respondent *m* entered the forest around the village in grid cell *i* more than once a month and ‘0’ when less than once a month.

### Dynamic abundance model

#### The model

We adapted a dynamic population model developed by Chandler & Clark^37^ for integrating count data and presence-absence data of a species. Our model generalizes the negative binomial model for open populations and assumes that abundance patterns are determined by an initial territory establishment process followed by gains and losses resulting from births, mortalities and dispersal. It also accounts for varying detection errors inherited from different survey data. Our model requires both spatial and temporal data and consists of four broad levels: (1) latent orangutan population density, (2) observed orangutan occurrence, (3) latent orangutan nest density, and (4) observed orangutan nest density and occurrence. The first level (latent orangutan population density) can be described as:

   *O*_*i*,*t*_ ~ Bernoulli(*φ*_*i*,*t*_)

   *Nou*_*i*,1_ ~ Poisson(*λ*_*i*_ × *O*_*i*,1_)

   *S*_*i*,*t*_ ~ Binomial(*Nou*_*i*,*t*−1_, *θ*_*i*,*t*_)

   *R*_*i*,*t*_ ~ Poisson(*δ*_*i*,*t*_)

   *Ñou*_*i*,*t*+1_ = *S*_*i*,*t*_ + *R*_*i*,*t*_

   *Nou*_*i*,*t*+1_ ~ Poisson(*Ñou*_*i*,*t*+1_ × *O*_*i*,*t*+1_)

The second level (observed orangutan occurrence) as:

   *Zou*_*i*,*m*,*t*_ ~ Bernoulli(*ρou*_*i*,*m*,*t*_ × *O*_*i*,*t*_)

The third level (latent orangutan nest density) as:

   *Nnest*_*i*,*t*_ = *ψ*_*i*,*t*_ × *Nou*_*i*,*t*_

Finally, the fourth level (observed orangutan nest density and occupancy) as:

   *Y*_*i*,*j*,*t*_ ~ Binomial(*Nnest*_*i*,*t*_, *ξ*_*i*,*j*,*t*_) for nest density

*Znest*_*i*,*k*,*t*_ ~ Bernoulli(*ρnest*_*i*,*k*,*t*_ × *Onest*_*i*,*t*_) for nest occupancy

where

*O*_*i*,*t*_ is the latent occurrence of orangutan at grid cell *i* in survey period *t*,

*Nou*_*i*,*t*_ is the latent number of orangutans at grid cell *i* in survey period *t*,

*S*_*i*,*t*_ is the latent number of survivors at grid cell *i* that do not emigrate between period *t* and *t* + 1,

*R*_*i*,*t*_ is the latent number of recruits (including births and immigrants) at grid cell *i* between period *t* and *t* + 1,

*Ñou*_*i*,*t*_ is the latent number of orangutans at grid cell *i* in survey period *t*, as a result of individuals survived and recruited in the previous survey period (*S*_*i*,*t*−*1*_ and *R*_*i*,*t*−*1*_, respecitively*)*,

*Zou*_*i*,*m*,*t*_ is the observed orangutan occurrence at grid cell *i* in survey period *t* from respondent *m*

*Nnest*_*i*,*t*_ is the latent number of orangutan nests at grid cell *i* in survey period *t*,

*Onest*_*i*,*t*_ is the latent occupancy of orangutan nests at grid cell *i* in survey period *t*, derived as a binary value of *Nnest*_*i*,*t*_

*Y*_*i*,*j*,*t*_ is the observed nest count at grid cell *i* in survey period *t* from survey type *j*,

*Znest*_*i*,*k*,*t*_ is the observed nest occurrence at sub-grid cell *k* and grid cell *i* in survey period *t*.

The parameters estimated from the model are the initial abundance rate at grid cell *i* (*λ*_*i*_), survival probability and recruitment rate at grid cell *i* between survey period *t* and *t* + 1 (*θ*_*i*,*t*_ and *δ*_*i*,*t*_), the orangutan occupancy rate at grid cell *i* and survey period *t* (*φ*_*i*,*t*_), the scaling factor of the nest and the orangutan density at grid cell *i* and survey period *t* (*ψ*_*i*,*t*_), the probability of detecting orangutan individuals from the interview survey at grid cell *i* and survey period *t* for respondent *m* (*ρou*_*i*,*m*,*t*_), the probability of detecting orangutan nests from the line transects at grid cell *i* and survey period *t* for survey type *j* (*ξ*_*i*,*j*,*t*_, where *j* ∈ {aerial, ground}), and the probability of detecting orangutan nests from the line transects and other targeted surveys at sub-grid cell *k* and grid cell *i* and survey period *t* (*ρnest*_*i*,*k*,*t*_).

These parameters can be modeled by including site-specific covariates. We modeled the initial abundance rate at grid cell *i*, i.e. *λ*_*i*_, as a function of altitude (*ALT*_*i*_), mean annual monthly rainfall during the dry season from May to September (*DRY*_*i*_), mean annual monthly rainfall during the dry season from November to March (*WET*_*i*_), the quadratic term of *ALT*_*i*_, *DRY*_*i*_ and *WET*_*i*_, nearest distance to protected areas (*DPA*_*i*,1_), the proportions of Muslims per district (*MS*_*i*_), natural forest extent (*FR*_*i*,1_), and the interaction between natural forest extent and nearest distance to forest recently converted to industrial agriculture (*FR*_*i*,1_ × *CFA*_*i*,1_) that all occurred prior to 2003, i.e.

1 $$\begin{array}{rcl}\mathrm{log}({\lambda }_{i}) & = & {\alpha }_{1}+{\alpha }_{2}AL{T}_{i}+{\alpha }_{3}AL{T}_{i}^{2}+{\alpha }_{4}DR{Y}_{i}+{\alpha }_{5}DR{Y}_{i}^{2}+{\alpha }_{6}WE{T}_{i}+{\alpha }_{7}WE{T}_{i}^{2}\\  &  & +{\alpha }_{8}DP{A}_{i,1}+{\alpha }_{9}M{S}_{i}+{\alpha }_{10}F{R}_{i,1}+{\alpha }_{11}(F{R}_{i,1}\times CF{A}_{i,1})\end{array}$$

Natural forest comprised mature natural forest cover that had not been completely cleared in the last 30 years^61^.

The occupancy rate and the survival rate at grid cell *i* between period *t*−1 and *t*, i.e. *φ*_*i*,*t*_ and *θ*_*i*,*t*_, respectively, were modeled in a similar manner as the initial abundance rate, i.e.

2 $$\begin{array}{rcl}{\rm{logit}}({\phi }_{i,t}) & = & {\beta }_{1}+{\beta }_{2}AL{T}_{i}+{\beta }_{3}AL{T}_{i}^{2}+{\beta }_{4}DR{Y}_{i}+{\beta }_{5}DR{Y}_{i}^{2}+{\beta }_{6}WE{T}_{i}\\  &  & +{\beta }_{7}WE{T}_{i}^{2}+{\beta }_{8}DP{A}_{i,t}+{\beta }_{9}M{S}_{i}+{\beta }_{10}F{R}_{i,t}+{\beta }_{11}(F{R}_{i,t}\times CF{A}_{i,t})\end{array}$$

3 $$\begin{array}{rcl}{\rm{logit}}({\theta }_{i,t}) & = & {\eta }_{1}+{\eta }_{2}AL{T}_{i}+{\eta }_{3}AL{T}_{i}^{2}+{\eta }_{4}DR{Y}_{i}+{\eta }_{5}DR{Y}_{i}^{2}+{\eta }_{6}WE{T}_{i}\\  &  & +{\eta }_{7}WE{T}_{i}^{2}+{\eta }_{8}DP{A}_{i,t}+{\eta }_{9}M{S}_{i}+{\eta }_{10}F{R}_{i,t}+{\eta }_{11}(F{R}_{i,t}\times CF{A}_{i,t})\end{array}$$

We included the quadratic term of *ALT*, *DRY* and *WET* to test the preference of orangutan to occupy areas with intermediate values for altitude and rainfall during the dry and wet season. We also tested whether or not proximity to protected areas (*DPA*) increases survival rates by reducing the risk of orangutan killings. Descriptions of the covariates used to explain the initial abundance, occupancy and survival rates are given in Supplementary Method 1.

The recruitment rate at grid cell *i* between period *t*−1 and *t*, i.e. *δ*_*i*,*t*_, was modeled as the number of individuals in site *i* and the neighboring sites at the previous survey period^62^, i.e.

4 $$\mathrm{log}({\delta }_{i,t})=\chi +\,\mathrm{log}(NEIG{H}_{i,t-1})\,{\rm{with}}\,NEIG{H}_{i,t-1}=\frac{1}{(|{n}_{i}|+1)}(\sum _{k\in {n}_{i}}{w}_{k}{N}_{k,t-1}+{N}_{i,t-1})$$

where *n*_*i*_ is the first-order neighbours surrounding grid cell *i* (Moore neighborhood) and *w*_*k*_ is a binary indicator (1 or 0) of whether grid cell *i* is connected to grid cell *k* ∈ *n*_*j*_. The binary indicator *w*_*k*_ was introduced to take into account the effect of large rivers on orangutan dispersal. We used a spatial map of the main rivers in Borneo and determined numerous rivers as barriers to orangutan dispersal, e.g. Kapuas, Barito, Kahayan, Katingan, Rungan, Lamandau, Landak, Mempawah, Mendawai, Paloh, Pawan, Seruyan, Mahakam, Kayan, Rajang, Baram and Kinabatangan. To build *w*_*k*_, we first constructed a vector of straight lines that connect the centre point of grid cell *i* and the centre point of each adjacent grid cell *k* ∈ *n*_*j*_^63^. This is to simulate the possible dispersal routes taken by an orangutan from grid cell *i* to the surrounding grid cells. We then intersected this line with the river barrier layer. We assumed *w*_*k*_ = 0 if at least one intersection was found within grid cell *k* ∈ *n*_*j*_ (i.e. rivers prevent orangutan dispersal from grid cell *i* to grid cell *k*) and *w*_*k*_ = 1 if no intersection was found.

In earlier studies, the density of orangutans at grid cell *i*, i.e. *gou*_*i*_, has typically been estimated by the following equation

5 $$go{u}_{i}=\frac{gnes{t}_{i}}{{b}_{i}\times {q}_{i}\times {d}_{i}}$$

where *b*_*i*_ is the proportion of nest builders, i.e. juveniles less than around 3 years of age are unlikely to build nests^64^, *q*_*i*_ is the daily rate of nest production, and *d*_*i*_ is the nest decay rate or the number of days a nest remains visible. Based on previous studies in Borneo, the proportion of nest builders has been estimated at around 0.9^4, 7, 23^. The average daily rate of nest production for Bornean orangutans has been estimated to range between 1 and 1.2^4, 7, 23^, but this can fluctuate depending on the level of forest disturbance, i.e. between primary and logged over forest^23^. Generally, the multiplication of *b*_*i*_ and *q*_*i*_ results in a value around 1. The nest decay rate is much more uncertain, however, ranging between 85 to over 800 days^21–23^ and has been shown to vary across different forest types and with altitude^4, 7, 23^. Hence, to take into account the variability in the total denominator of Eq. (5) across different grid cells *i* and survey periods *t*, we modeled *ψ*_*i*,*t*_ as

6 $${\psi }_{i,t}=100\times ({\gamma }_{0}+{\gamma }_{1}MG{V}_{i,t}+{\gamma }_{2}P{T}_{i,t}+{\gamma }_{3}LOW{L}_{i,t}+{\gamma }_{4}MON{T}_{i,t}+{\gamma }_{5}FRG{M}_{i,t})$$

where *MGV*_*i*,*t*_ is a binary variable denoting whether or not the majority of forest at grid cell *i* and time *t* are mangrove forest, and similarly *PT*_*i*,*t*_ for peat forest, *LOWL*_*i*,*t*_ for lowland forest (altitude < 500 m), *MONT*_*i*,*t*_ for montane forest (altitude ≥ 500 m), and *FRGM*_*i*,*t*_ for highly fragmented forest (< 25 ha per km^2^).

The probability of detecting orangutans from the interview surveys at grid cell *i* and time *t* for respondent *m*, i.e. *ρou*_*i*,*m*,*t*_, was modeled as a function of respondents’ frequency for entering the forest around the village (1 for more than once a month and 0 for less than once a month), i.e. *FE*_*i*,*m*_, such that

7 $${\rm{logit}}(\rho o{u}_{i,m,t})={\upsilon }_{1}+{\upsilon }_{2}F{E}_{i,m}$$

The probability of detecting orangutan nests at grid cell *i* and time *t* and for survey *j* (*j* ∈ {aerial, ground}), i.e. *ξ*_*i*,*j*,*t*_, was modeled constant for each survey type, such that

8 $${\rm{logit}}({\xi }_{i,j,t})={\mu }_{j}$$

Finally, the probability of detecting orangutan nests at sub-grid cell *k* and grid cell *i* and time *t* for line transects and other targeted surveys, i.e. *ρnest*_*i*,*k*,*t*_, was modeled constant, such that

9 $${\rm{logit}}(\rho nes{t}_{i,k,t})=\zeta $$

#### Model fitting and evaluation

We used WinBUGS Version 1.4.3^65^ to estimate the parameter posterior distributions and the regression coefficients for *λ*_*i*_, *φ*_*i*,*t*_, *θ*_*i*,*t*_, *δ*_*i*,*t*_, *ψ*_*i*,*t*_, *ρou*_*i*,*m*,*t*_, *ξ*_*i*,*j*,*t*_, and *ρnest*_*i*,*k*,*t*_. The WinBUGS code for the dynamic abundance model is provided in Supplementary Method 2. We assumed a vague prior for each parameter, as described in Table 2.

We ran three Markov chain Monte Carlo (MCMC) chains, where each chain consists of 100,000 iterations and the first 50,000 were discarded as burn-in. To improve convergence and to reduce the autocorrelation in the MCMC chain, we standardized all variables prior to model fitting. Prior to fitting the model to the data, we tested the correlation among the original (unstandardized) environmental variables explaining *λ*, *φ*_*t*_ and *θ*_*t*_, i.e. variables *ALT*, *DRY*, *WET*, *DPA*, *MS*, *FR* and *CFA*, and also among the standardized variables. Convergence for each model parameter was assessed from the values of Rhat statistics and visualization of the chain plot of the MCMC iterations. Rhat values around 1 and the absence of seasonality within each chain plot and overlap among the chains indicate convergence. We also tested for correlations among posterior distributions of the coefficients, especially between the linear and the quadratic terms of variables *ALT*, *DRY* and *WET*, to ensure correct functional forms were specified for these variables and the coefficients were not biased.

The goodness-of-fit of the model was assessed by comparing the simulated nest abundance predictions for each time period with the observed nest counts. For each simulated prediction and time period, we calculated the Pearson’s correlation coefficient *r* and also fitted a linear regression between the predicted values and the observed values to calculate the *R* ^2^ value^66^. We also validated the simulated orangutan presence-absence predictions for each time period against the actual observations based on interview surveys. In the validation dataset, we defined “presence” in a village if at least one respondent reported the occurrence of orangutan, and we defined “absence” if more than 50% of the respondents who enter the forest more than once a month had never seen the species. We used the proportions of correctly predicted presence or Sensitivity (SN) and the proportions of correctly predicted absence or Specifity (SP) as the measure of performance. SN and SP values close to one indicate high accuracy.

#### Assessing orangutan abundance change among regions and land uses

We assessed orangutan population trends by measuring the change in the number of individuals obtained from the simulated predictions. We investigated how the trends vary across different regions (states and provinces), as well as across different land uses. We considered five land use categories: (1) protected areas (PA), (2) logging concessions on natural forests (LOGG), (3) industrial timber plantation concessions (ITP), (4) oil palm plantation concessions (OPP), and (5) outside protected areas, infrastructure and urban areas and without concessions, mostly small-scale agriculture and smaller forest patches (OTHER). We obtained spatial boundary data for protected areas, logging concessions, timber plantation concessions, and oil palm concessions for Kalimantan, Sabah and Sarawak for 2000, 2006 and 2012 from various sources (see Supplementary Method 3).

#### Assessing drivers of orangutan population decline among regions and land uses

To inform orangutan conservation planning, we assessed the drivers of orangutan population decline in each region. This was achieved mainly by relating the environmental covariates explaining survival rates in Eq. (3) across 1 × 1 km^2^ grid cells where orangutans are predicted to occur with known actual threats observed on Borneo. These threats includes: (1) habitat loss, i.e. the loss of natural forest of orangutan habitats, (2) human-orangutan conflicts, (3) anthropogenic human activities, such as hunting and poaching, and (4) habitat fragmentation, i.e. breaking up intact forest habitats into small forest patches.

The decline of orangutan population due to habitat loss in grid cell *i* at time period *t*, i.e. *HLOSS*_*i*,*t*_, was related specifically to forest cover covariate *FR*_*i*,*t*_ (i.e. the 10^th^ additive component in Eq. (3)). We measured habitat loss based on counterfactual analysis, i.e. the discrepancy between the survival rates under the ‘counterfactual assumption of no forest loss, or forest cover remains the same as in the previous time period (*FR*_*i*,*t*−*1*_)’ versus ‘the actual forest cover in that period (*FR*_*i*,*t*_)’, such that

$$HLOS{S}_{i,t}=F{R}_{i,t-1}-F{R}_{i,t}$$

High *HLOSS*_*i*,*t*_ implies low orangutan survival rate, or high contribution of habitat loss to population decline in grid cell *i* at time period *t*.

The decline of orangutan population due to human-orangutan conflicts in grid cell *i* at time period *t*, i.e. *CONFL*_*i*,*t*_, was related specifically to the interaction between forest cover *FR*_*i*,*t*_ and the distance to newly converted forest to industrial agriculture *CFA*_*i*,*t*_ (i.e. the 11^th^ additive component in Eq. (3)), such that

$$CONF{L}_{i,t}=F{R}_{i,t}\times CF{A}_{i,t}$$

Low *CONFL*_*i*,*t*_ implies low orangutan survival rate, or high contribution of human-orangutan conflicts to population decline in grid cell *i* at time period *t*.

For measuring the decline of orangutan population due to anthropogenic activities in grid cell *i* at time period *t*, i.e. *ANTH*_*i*,*t*_, we used monthly rainfall during the dry *DRY*_*i*_ and the wet seasons *WET*_*i*_ and proximity to protected areas *DPA*_*i*,*t*_ as proxy (i.e. 4–8^th^ additive components in Eq. (3)), such that

$$ANT{H}_{i,t}={\hat{\eta }}_{4}DR{Y}_{i}+{\hat{\eta }}_{5}DR{{Y}_{i}}^{2}+{\hat{\eta }}_{6}WE{T}_{i}+{\hat{\eta }}_{7}WE{{T}_{i}}^{2}+{\hat{\eta }}_{8}DP{A}_{i,t}$$

where ${\hat{\eta }}_{4},{\hat{\eta }}_{5},{\hat{\eta }}_{6},{\hat{\eta }}_{7},\,{\rm{and}}\,{\hat{\eta }}_{8}$ are the estimated coefficients obtained from WinBUGS simulations. This is because seasonal rainfall patterns determine socio-economic structure and livelihoods on Borneo^34^. Additionally, protected areas were assumed to provide a refuge for the species against hunting and poaching^11^. Low *ANTH*_*i*,*t*_ implies low orangutan survival rate, or high contribution of anthropogenic activities to population decline in grid cell *i* at time period *t*.

To obtain the relative influence of habitat loss as a driver of orangutan population decline for each region, we averaged *HLOSS*_*i*,*t*_ across all grid cells where orangutans are predicted to occur within the respective region. To obtain the relative influence of human-orangutan conflicts and anthropogenic activities as drivers of population decline for each region, we applied similar procedure to *CONFL*_*i*,*t*_ and *ANTH*_*i*,*t*_, respectively. We also assessed how these drivers vary across different land uses (i.e. LOGG, ITP, OPP and OTHER) within each region.

For habitat fragmentation, we assessed this as a driver over the entire orangutan distribution range across different landscapes within the region. Because territorial ranges of orangutans, especially the females, are generally restricted to a maximum of 850 ha^55^, the species’ dispersal opportunities between habitat fragments are generally limited. This implies that landscapes with isolated forest patches of orangutan habitats (i.e. fragmented habitats) have a higher risk of orangutan decline due to lower colonization rates than landscapes with better habitat connectivity. The relative influence of habitat fragmentation as a driver of orangutan population decline in a region, i.e. *FRAG*, was estimated as the interaction between the mean size of contiguous forest where orangutan occurred and the mean reciprocal distance of each contiguous forest to the nearest forest patch. Low *FRAG* implies low orangutan survival rate, or high contribution of habitat fragmentation to population decline.

## Electronic supplementary material

Supplementary Information
