# Supplementary material for: First integrative trend analysis for a great ape species in Borneo
Source: Sci Rep. 2017 Jul 7;7:4839. doi: 10.1038/s41598-017-04435-9 (PMC5501861; doi:10.1038/s41598-017-04435-9)
Supplement: Supplementary file 1 — Supplementary Information [file 41598_2017_4435_MOESM1_ESM.doc]

First integrative trend analysis for a great ape species in Borneo

Truly Santika, Marc Ancrenaz, Kerrie A. Wilson, Stephanie Spehar, Nicola Abram, Graham L. Banes, Gail Campbell-Smith, Lisa Curran, Laura d'Arcy, Roberto A. Delgado, Andi Erman, Benoit Goossens, Herlina Hartanto, Max Houghton, Simon J. Husson, Hjalmar Kuehl, Isabelle Lackman,

Ashley Leiman, Karmele Llano Sanchez, Niel Makinuddin, Andrew J. Marshall, Ari Meididit,

Kerrie Mengersen, Musnanda, Nardiyono, Anton Nurcahyo, Kisar Odom, Adventus Panda,

Didik Prasetyo, Purnomo, Andjar Rafiastanto, Slamet Raharjo, Dessy Ratnasari, Anne E. Russon,

Adi H. Santana, Eddy Santoso, Iman Sapari, Jamartin Sihite, Ahmat Suyoko, Albertus Tjiu,

Sri Suci Utami-Atmoko, Carel P. van Schaik, Maria Voigt, Jessie Wells, Serge A. Wich,

Erik P. Willems & Erik Meijaard

**Supplementary Table 1**

Correlations among environmental variables

**Supplementary Table 1.** Pearson correlations among the standardized environmental variables explaining *λ*, *φ_t_* and *θ_t_* in Eq. (1)-(3) for time period *t*, i.e. variables *ALT*, *DRY*, *WET*, *DPA_t_*, *MS*, *FR_t_* and *CFA_t_*.

| Time period | First variable | Second variable | | | | | |
| --- | --- | --- | --- | --- | --- | --- | --- |
|  |  |  |  |  |  |  |  |
|  |  | ALT | DRY | WET | DPA_1_ | MS | FR_1_ |
| 1997-2002 | *DRY* | 0.332 |  |  |  |  |  |
|  | *WET* | -0.059 | 0.352 |  |  |  |  |
|  | *DPA*_1_ | 0.211 | 0.182 | 0.217 |  |  |  |
|  | *MS* | -0.272 | -0.360 | -0.336 | 0.168 |  |  |
|  | *FR*_1_ | 0.362 | 0.370 | 0.143 | 0.313 | -0.157 |  |
|  | *CFA*_1_ | 0.261 | 0.289 | 0.236 | 0.281 | -0.210 | 0.350 |
|  |  |  |  |  |  |  |  |
|  |  | ALT | DRY | WET | DPA_2_ | MS | FR_2_ |
| 2003-2008 | *DRY* | 0.332 |  |  |  |  |  |
|  | *WET* | -0.059 | 0.352 |  |  |  |  |
|  | *DPA*_2_ | 0.189 | 0.179 | 0.221 |  |  |  |
|  | *MS* | -0.272 | -0.360 | -0.336 | 0.171 |  |  |
|  | *FR*_2_ | 0.410 | 0.321 | 0.138 | 0.285 | -0.154 |  |
|  | *CFA*_2_ | 0.384 | 0.339 | 0.084 | 0.219 | -0.147 | 0.402 |
|  |  |  |  |  |  |  |  |
|  |  | ALT | DRY | WET | DPA_3_ | MS | FR_3_ |
| 2009-2015 | *DRY* | 0.332 |  |  |  |  |  |
|  | *WET* | -0.059 | 0.352 |  |  |  |  |
|  | *DPA*_3_ | 0.181 | 0.192 | 0.210 |  |  |  |
|  | *MS* | -0.272 | -0.360 | -0.336 | 0.169 |  |  |
|  | *FR*_3_ | 0.433 | 0.363 | 0.121 | 0.214 | -0.149 |  |
|  | *CFA*_3_ | 0.335 | 0.295 | 0.065 | 0.195 | -0.068 | 0.419 |
|  |  |  |  |  |  |  |  |

**Supplementary Table 2**

Predicted density and decline rate of orangutans by region

**Supplementary Table 2.** Estimated mean density of orangutan individuals per 100 km^2^ per time period between 1997 and 2015, and the 95% credible interval (CI) for the mean (a), and the rate of population decline by region (b).

| Region |  | (a) Estmated density  (individuals per 100 km^2^) | | | | | | | |  | (b) Decline rate | |
| --- | --- | --- | --- | --- | --- | --- | --- | --- | --- | --- | --- | --- |
|  |  | 1997-2002 | |  | 2003-2008 | |  | 2009-2015 | |  | Over 12 years ‡ | Adjusted  per 10 years |
|  |  | Mean (CI) | |  | Mean (CI) | |  | Mean(CI) | |  |  |  |
| *Sabah* |  | 20 | (15,24) |  | 17 | (13,21) |  | 15 | (11,18) |  | 25.5% | 21.3% |
| *Sarawak* |  | 2 | (1,2) |  | 1 | (1,2) |  | 1 | (1,2) |  | 26.6% | 22.2% |
| *West Kalimantan* |  | 19 | (15,23) |  | 15 | (12,19) |  | 12 | (9,15) |  | 35.3% | 29.4% |
| *Central Kalimantan* |  | 32 | (26,39) |  | 27 | (21,33) |  | 23 | (18,27) |  | 29.9% | 24.9% |
| *East Kalimantan* |  | 6 | (5,7) |  | 5 | (4,6) |  | 4 | (3,5) |  | 26.9% | 22.4% |
| *North Kalimantan* |  | 1 | (1,1) |  | 1 | (1,1) |  | 1 | (1,1) |  | 18.4% | 15.3% |
| ***Overall*** |  | **15** | **(12,18)** |  | **12** | **(10,15)** |  | **10** | **(8,12)** |  | **30.3%** | **25.3%** |

‡ based on the median value of the first time period (1997-2002, median 2000) and the third period (2009-2015, median 2012)

**Supplementary Figure 1**

Trace plots of the MCMC iterations


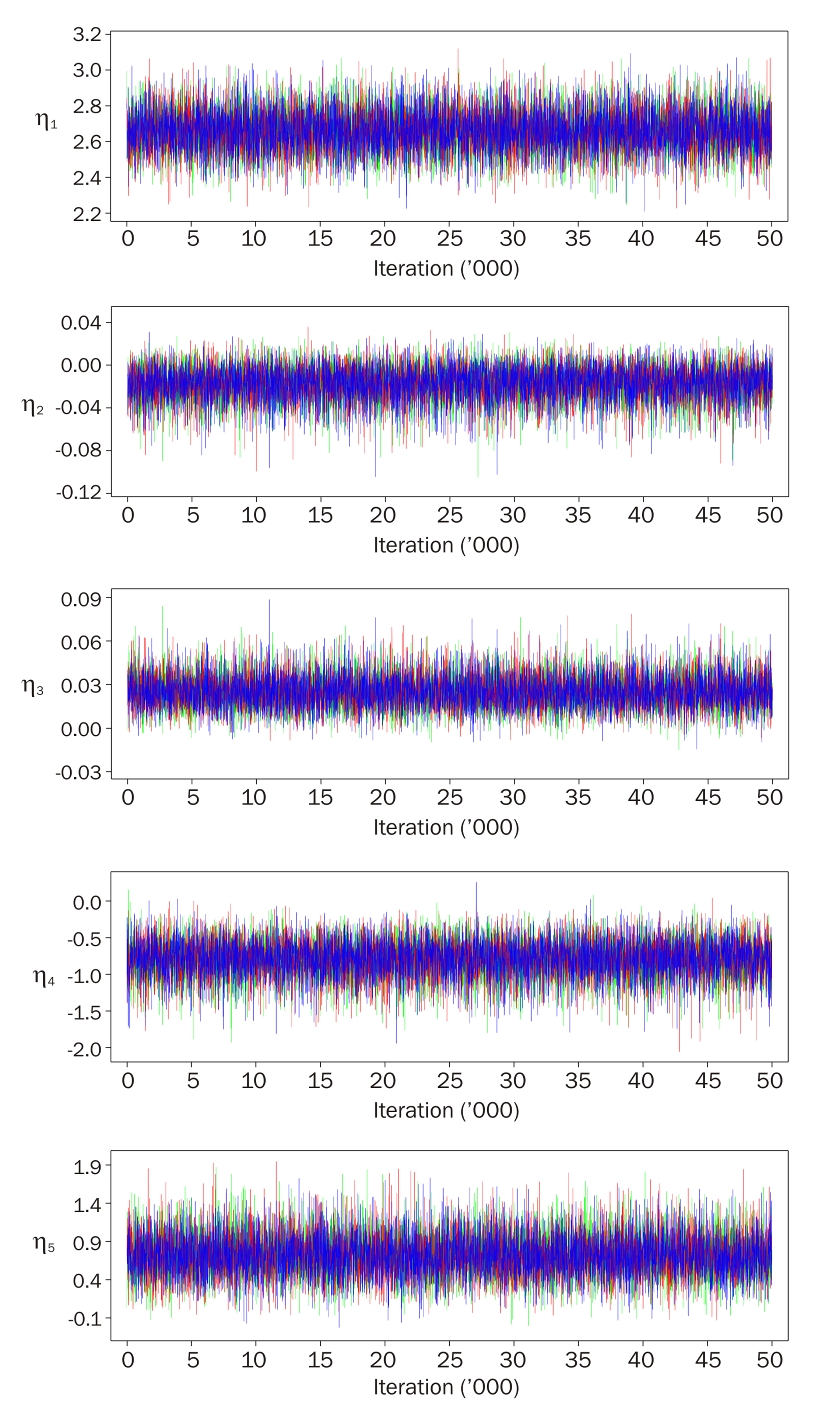


**Supplementary Figure 1.** Trace plots of the MCMC iterations for variables explaining survival rates (*θ*), including: (a) the intercept (*η*_1_), (b) *ALT* (*η*_2_), (c) *ALT*^2^ (*η*_3_), (d) *DRY* (*η*_4_), and (e) *DRY*^2^ (*η*_5_), showing the absence of seasonality, which indicates convergence. Similar results were obtained for other variables in the model.

**Supplementary Figure 2**

Correlations between posterior distributions of the coefficients of the linear and quadratic terms for *ALT*, *DRY* and *WET*


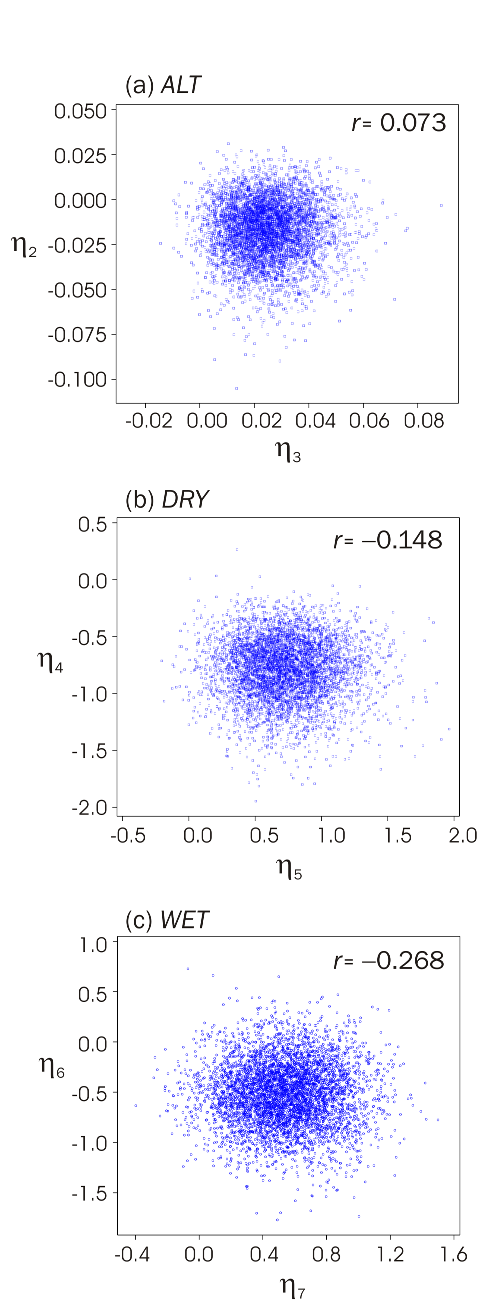


**Supplementary Figure 2.** Scatter plots of the relationship between the posterior distributions of the coefficients of the linear and quadratic terms for variables explaining survival rates (*θ*), including: (a) *ALT* (*η*_2_ against *η*_3_), (b) *DRY* (*η*_4_ against *η*_5_), and (c) *WET* (*η*_6_ against *η*_7_), showing weak correlations between the linear and quadratic terms. Similar results were obtained for the respective variables explaining the initial abundance (*λ*) and occupancy rates (*φ*).

**Supplementary Figure 3**

Comparison between historical population estimates and our trend model estimates


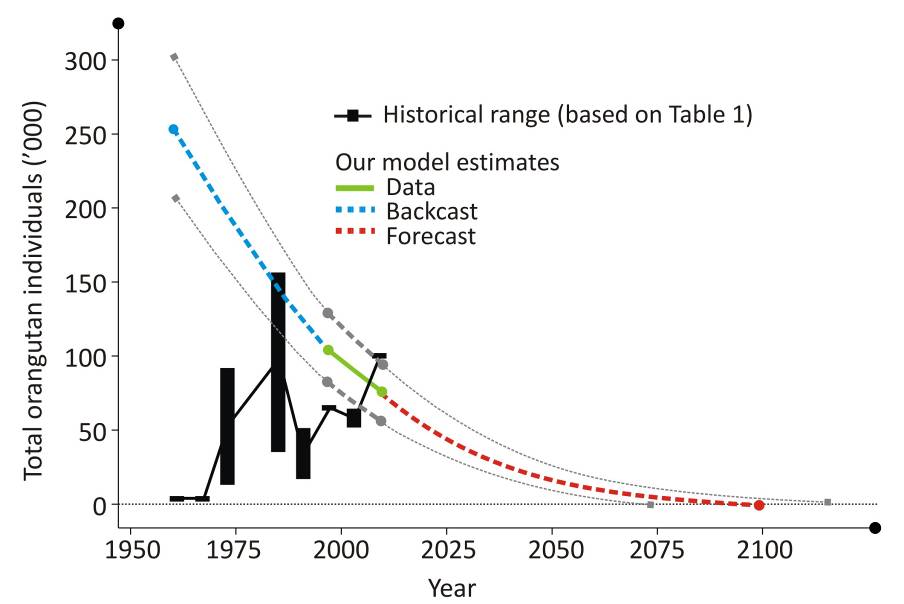


**Supplementary Figure 3.** Comparison between historical population estimates and the trend predicted by our model. Historical population estimates show an increasing trend through time due to increasing availability of survey data. See Table 1 for historical data.

**Supplementary Figure 4**

Altitude zones, long-term annual rainfall zones, and intermediate rainfall zones on Borneo


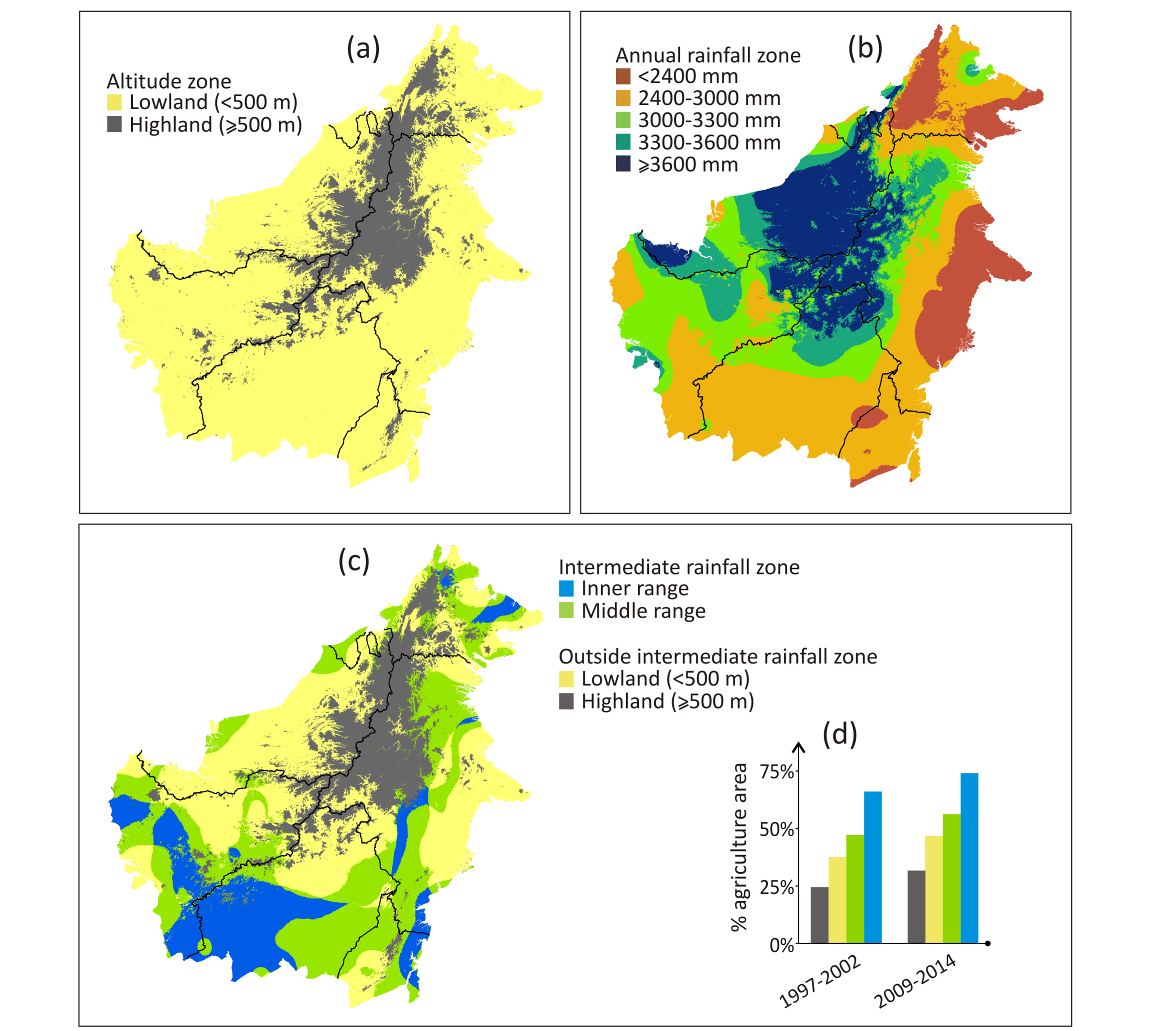


**Supplementary Figure 4.** Altitude zones on Borneo (i.e. lowland (<500 m) and highland (≥500 m)) derived from the SRTM digital elevation data ^1^ (a). Long-term annual rainfall zones, derived from the WorldClim Global Climate Data ^2^ (b). Intermediate rainfall zones (which include inner range and middle range, representing area that are close and moderately close to intermediate rainfall that is optimal for plant productivity and agriculture (i.e. 150-250 mm per month during the dry season and 200-400 mm per month during the wet season)), lowlands outside the intermediate rainfall zone, and highland, derived from the SRTM data ^1^ and WorldClim ^2^ (c), with the proportion of agriculture area (plantations and agriculture fields and shrublands from abandoned agriculture) outside the government sanctioned protected areas during 1997-2002 and 2009-2015 within each zone in (d). These maps are available at https://figshare.com/s/2b35a6caa8c2ea2c59aa ^3^.

**References for Supplementary Figure 4**

1. Jarvis, A., Reuter, H.I., Nelson, A. & Guevara, E. *Hole-filled SRTM for the Globe Version 4* (2008)*.* Available at http://srtm. csi. cgiar. org (accessed 21 December 2015).
2. Hijmans, R.J., Cameron, S.E., Parra, J.L., Jones, P.G. & Jarvis, A. 2005. Very high resolution interpolated climate surfaces for global land areas. *Int. J. Climatol*. **25**, 1965-1978 (2005). Available at http://www.worldclim.org/version1 (accessed 15 September 2015).
3. Santika, T. *Climate and altitude zones on Borneo*. figshare (2016). Available at https://figshare.com/s/2b35a6caa8c2ea2c59aa.

**Supplementary Figure 5**

The relationship between the proportion of area within district that overlap with intermediate rainfall zone and socio-economic variables


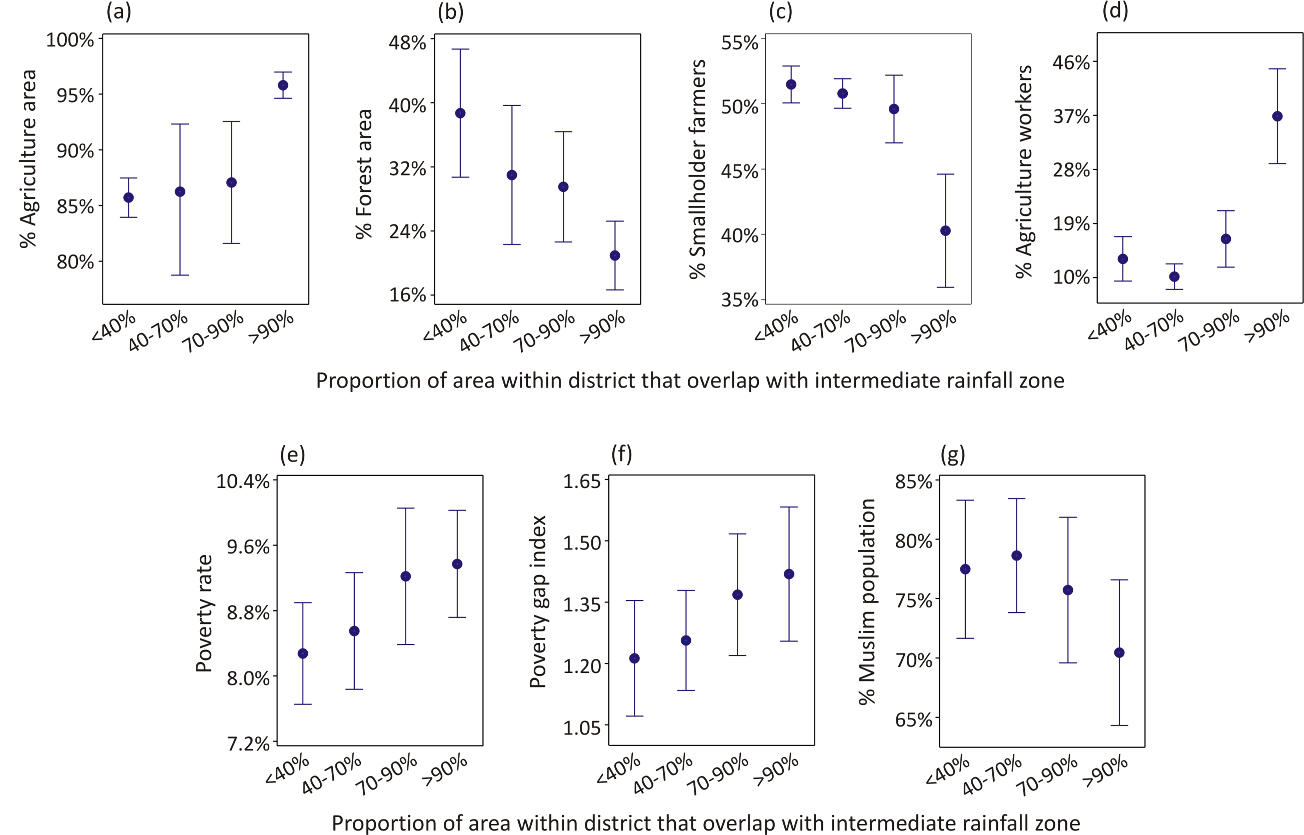


**Supplementary Figure 5.** The relationship between the proportion of area within district that overlap with intermediate rainfall zone, i.e. inner and middle range shown in Supplementary Figure 4 and the proportion of agriculture area (a), proportion of forest area (b), proportion of smallholder farmers (c), proportion of workers engaged in agriculture activities (either fully or temporarily employed, mainly by large agriculture companies) (d), percentage of local communities living under poverty (e), poverty gap index (f), and the proportion of Muslim population within the district (g).

**Supplementary Figure 6**

Habitat connectivity across different regions on Borneo


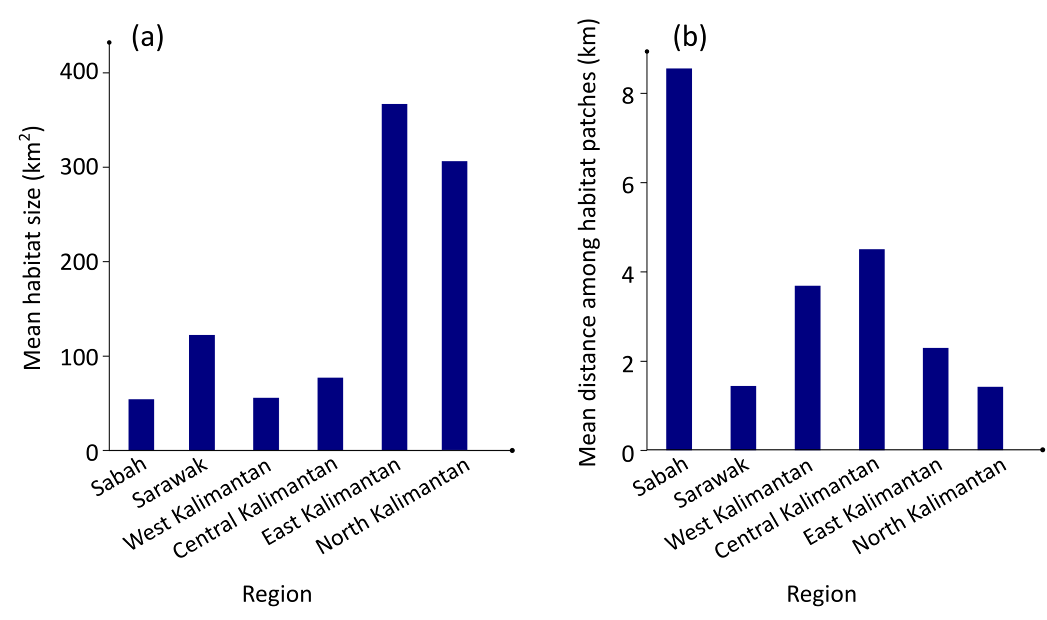


**Supplementary Figure 6.** The mean size of contiguous forest of orangutan habitats (a) and the mean distance among forest patches (b), across different regions on Borneo.

**Supplementary Method 1**

Covariates used to explain the parameters in the dynamic abundance model

We used a set of variables to explain the initial abundance (*λ*), occupancy rate (*φ*) and survival rate (*θ*) parameters. These include static variables altitude (*ALT*), mean annual monthly rainfall during the dry season from May to September (*DRY*), mean annual monthly rainfall during the dry season from November to March (*WET*) and the proportion of Muslim populations within districts (*MS*) (Fig. M1.1); and dynamic variables natural forest extent (*FR*), nearest distance to newly converted forest to industrial agriculture (*CFA*), and nearest distance to protected areas (*DPA*) (Fig. M1.2a-c). We used dynamic forest types to explain the scaling factor of nest and orangutan densities (*ψ*), where forest types comprise: (1) mangrove forest (*MGV*), (2) peat forest (*PT*), (3) lowland forest (altitude <500 m) (*LOWL*), (4) montane forest (altitude ≥500 m) (*MONT*) and (5) highly fragmented forest (<25 ha per km^2^) (*FRGM*) (Fig. M1.2d). Sources of data used to generate these variables are given in Table M1. Maps of the static variables are available at https://figshare.com/s/0f8319c7ae9e64482270 ^1^, and maps of the dynamic variables are available at https://figshare.com/s/18dea7404571149da148 ^2^.


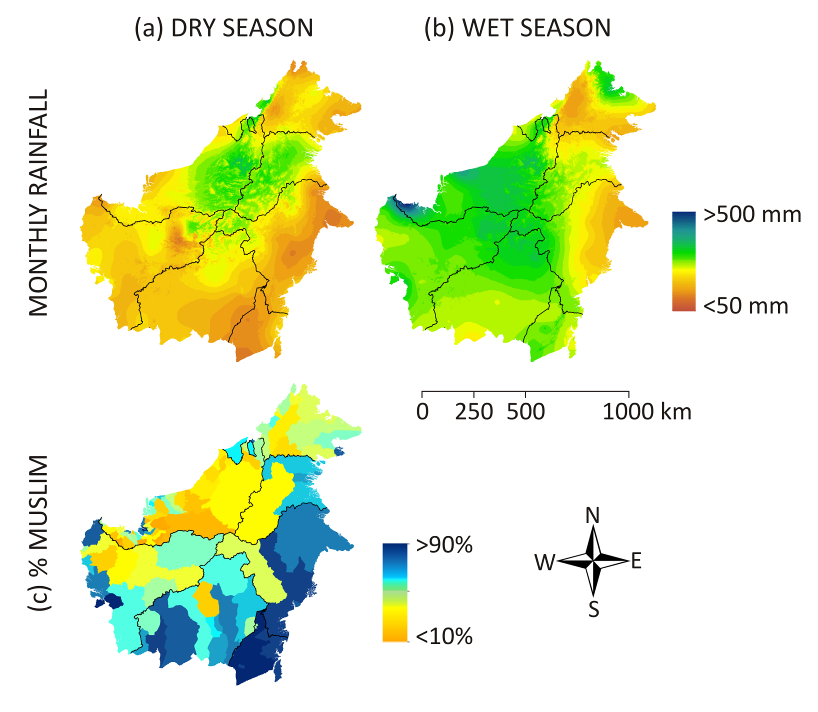


**Fig. M1.1.** The mean annual monthly rainfall during the dry season (May to September) (*DRY*) (a), the mean annual monthly rainfall during the wet season (November to March) (*WET*) (b), derived from the WorldClim Global Climate Data ^3^, and the proportion of Muslim populations at district level (*MS*) derived from the Indonesian Population Census 2010 ^4^ (c). Black lines indicate the regional boundaries (i.e. national, state, and provincial boundaries). These maps are available at https://figshare.com/s/0f8319c7ae9e64482270 ^1^.


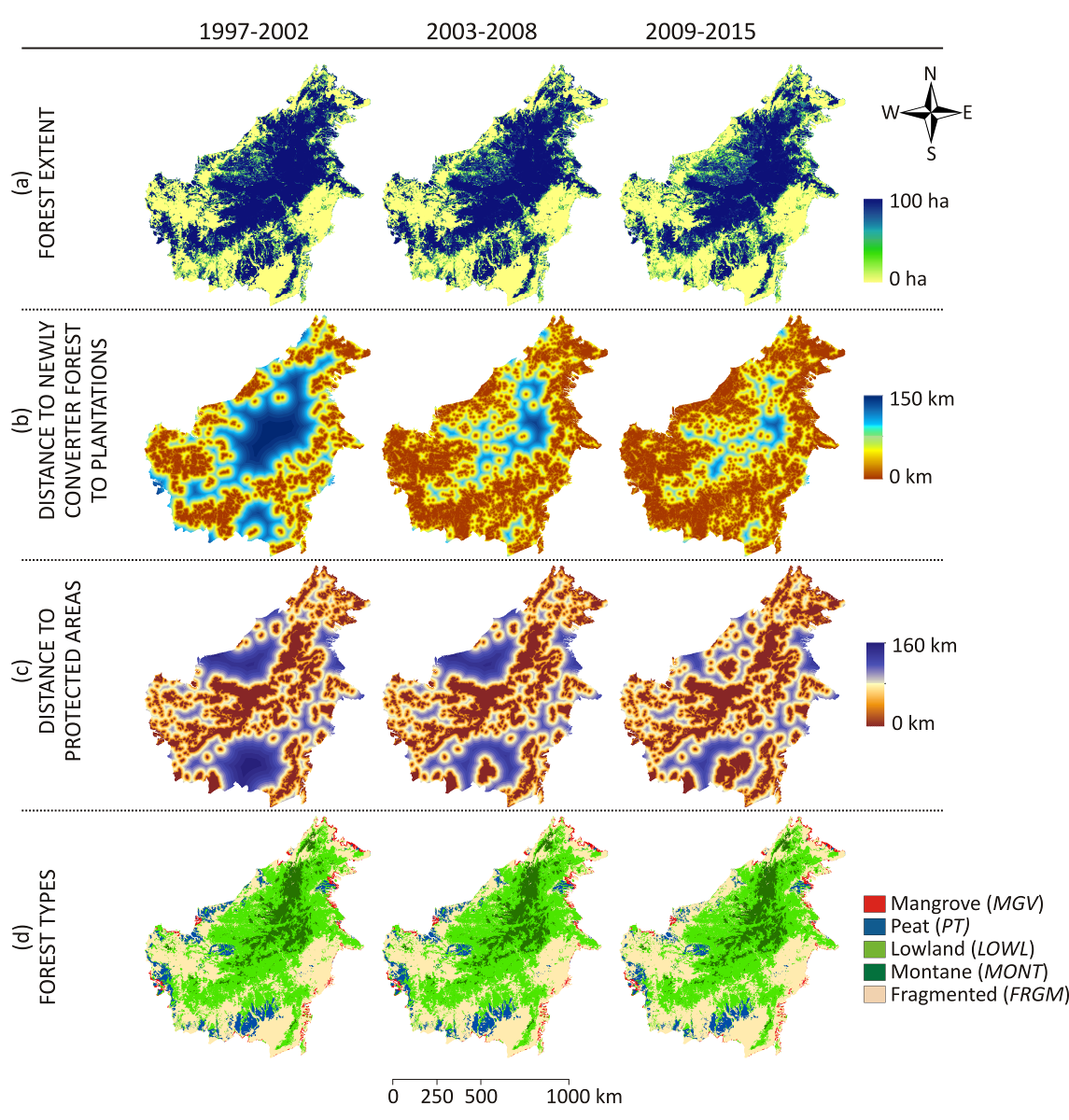


**Fig. M1.2.** The extent of natural forest per km^2^ (*FR*) derived from the Global Forest Change data ^5^ and data on the extent of Primary and Secondary forest provided by the Ministry of Environment and Forestry Indonesia ^6^ (a), the mean nearest distance to newly converted forest to industrial agriculture (*CFA*) derived from the Global Forest Change data and Land Cover data provided by the Ministry of Environment and Forestry Indonesia ^6^ (b), the mean nearest distance to protected areas (*DPA*) derived from Forest Functional Zone map provided by the Ministry of Environment and Forestry Indonesia ^7^ (c), and the majority of forest type derived from Land Cover data provided by the Ministry of Environment and Forestry Indonesia ^6^ (d), on Borneo in three time periods between 1997 and 2015. These maps are available at https://figshare.com/s/18dea7404571149da148 ^2^.

**Table M1.** Sources of data used to build the variables explaining the initial abundance (*λ*), occupancy rate (*φ*), survival rate (*θ*), and the scaling factor of nest and orangutan densities (*ψ*) parameters.

| Variable | Description | Type of variable | Static/  Dynamic | | | Data sources | |
| --- | --- | --- | --- | --- | --- | --- | --- |
|  |  |  |  | | |  | |
| *ALT* | Altitude | Continuous (m) | Static | | | 8 | |
| *DRY* | Long-term mean monthly rainfall during the dry season | Continuous (mm) | Static | | | 3 | |
| *WET* | Long-term mean monthly rainfall during the wet season | Continuous (mm) | Static | | | 3 | |
| *MS* | Proportions of Muslim populations within district | Continuous (%) | Static | | | 4 | |
| *FR* | Natural forest extent | Continuous (ha) | Dynamic  (1997-2002, 2003-2008, 2009-2015) | | | 5, 6 | |
| *CFA* | Nearest distance to newly converted forest to industrial agriculture | Continuous (log(km)) | Dynamic  (1997-2002, 2003-2008, 2009-2015) | | | 5, 6 | |
| *DPA* | Distance to protected areas | Continuous (log(km)) | Dynamic  (1997-2002, 2003-2008, 2009-2015) | | | 7 | |
| *MGV* | Location on mangrove forest | Binary | Dynamic  (1997-2002, 2003-2008, 2009-2015) | | | 6 | |
| *PT* | Location on peat forest | Binary | Dynamic  (1997-2002, 2003-2008, 2009-2015) | | | 6 | |
| *LOWL* | Location on lowland forest (<500 m) | Binary | Dynamic  (1997-2002, 2003-2008, 2009-2015) | | | 6 | |
| *MONT* | Location on montane forest  (≥ 500 m) | Binary | Dynamic  (1997-2002, 2003-2008, 2009-2015) | | | 6 | |
| *FRGM* | Location on fragmented forest (<25 ha per km^2^) | Binary | Dynamic  (1997-2002, 2003-2008, 2009-2015) | | | 6 | |
|  |  |  | |  |  | |  |

**References for Supplementary Method 1**

1. Santika, T. *Seasonal precipitation and Muslim populations on Borneo*. figshare (2016). Available at https://figshare.com/s/0f8319c7ae9e64482270.
2. Santika, T. *Dynamic Environment on Borneo*. figshare (2016). Available at https://figshare.com/s/18dea7404571149da148.
3. Hijmans, R.J., Cameron, S.E., Parra, J.L., Jones, P.G. & Jarvis, A. 2005. Very high resolution interpolated climate surfaces for global land areas. *Int. J. Climatol*. **25**, 1965-1978 (2005). Available at http://www.worldclim.org/version1 (accessed 15 September 2015).
4. Bureau of Statistics (BPS) Indonesia *Population Census 2010* (Jakarta, Indonesia 2011). Available at http://sp2010.bps.go.id (accessed 10 September 2015).
5. Hansen, M.C. *et al*. High-resolution global maps of 21st-century forest cover change. *Science* **342**, 850-853 (2013). Available at https://earthenginepartners.appspot.com/science-2013-global-forest (accessed 20 September 2015).
6. Ministry of Environment and Forestry. *Peta Penutupan Lahan* (Land Cover Map) Forest Planology Agency, Ministry of Environment and Forestry, Jakarta, Indonesia, 2000, 2006, 2012). Available at http://webgis.dephut.go.id:8080/kemenhut/index.php/id/fitur/layanan (accessed 21 March 2016).
7. Ministry of Environment and Forestry. *Peta Kawasan Hutan* (Forest Functional Zone Map) Forest Planology Agency, Ministry of Environment and Forestry, Jakarta, Indonesia, 2013). Available at http://webgis.dephut.go.id:8080/kemenhut/index.php/id/fitur/layanan (accessed 25 March 2016).
8. Jarvis, A., Reuter, H.I., Nelson, A. & Guevara, E. *Hole-filled SRTM for the Globe Version 4* (2008)*.* Available at http://srtm. csi. cgiar. org (accessed 21 December 2015).

**Supplementary Method 2**

WinBUGS code

#=============================================================================

# First integrative trend analysis for a great ape species in Borneo

# Authors: Truly Santika, Marc Ancrenaz, Kerrie A. Wilson, et al.

#=============================================================================

# WINBUGS code (core component)

OuBorneoPopModel = "model

{

# PRIORS

for (i in 1:11)

{

alpha[i] ~ dunif(-8,8)

beta[i] ~ dunif(-6,6)

eta[i] ~ dunif(-4,4)

}

for (i in 1:2)

{

upsilon[i] ~ dunif(-4,4)

}

gamma0 ~ dunif(0,10)

for (i in 1:5)

{

gamma1[i] ~ dunif(-10,10)

}

for (i in 1:2)

{

mu[i] ~ dunif(-4,4)

}

zeta ~ dunif(-4,4)

chi ~ dunif(-6,6)

# INITIAL ABUNDANCE (TIME PERIOD 1)

for (i in 1:I)

{

# LEVEL 1 (LATENT ORANGUTAN POPULATION)

# Latent abundance and distribution

log(lambda[i,1]) <- alpha[1]+alpha[2]*ALT[i]+ alpha[3]*pow(ALT[i],2) +alpha[4]*DRY[i]+alpha[5]*pow(DRY[i],2)+alpha[6]*WET[i] +alpha[7]*pow(WET[i],2)+alpha[8]*DPA[i,1]+alpha[9]*MS[i]

+alpha[10]*FR[i,1]+alpha[11]*(FR[i,1]*CFA[i,1])

logit(phi[i,1]) <- beta[1]+beta[2]*ALT[i]+ beta[3]*pow(ALT[i],2) +beta[4]*DRY[i]+beta[5]*pow(DRY[i],2)+beta[6]*WET[i] +beta[7]*pow(WET[i],2)+beta[8]*DPA[i,1]+beta[9]*MS[i]

+beta[10]*FR[i,1]+beta[11]*(FR[i,1]*CFA[i,1])

O[i,1] ~ dbern(phi[i,1])

Nu[i,1] <- lambda[i,1]*O[i,1]

N.ou[i,1] ~ dpois(Nu[i,1])

# LEVEL 2 (ORANGUTAN OBSERVATION)

# Observed/unobserved orangutan data from interview survey

for (m in 1:M)

{

logit(rhoou[i,m,1]) <- upsilon[1] + upsilon[2]*FE[i,m]

zi[i,m,1] <- rhoou[i,m,1]*O[i,1]

Z.ou[i,m,1] ~ dbern(zi[i,m,1]) # Occurrence data

}

# LEVEL 3 (LATENT ORANGUTAN NEST POPULATION)

# Abundance and nest relationship

psi[i,1] <- gamma0+gamma1[1]*MGV[i,1]+gamma1[2]*PT[i,1]+ gamma1[3]*LOWL[i,1]+gamma1[4]*MONT[i,1]+gamma1[5]*FRGM[i,1]

N.nest[i,1] ~ dpois(N.ou[i,1]*psi[i,1]*100)

O.nest[i,1] <- step(N.nest[i,1]-1)

# LEVEL 4 (NEST OBSERVATION)

# Count data for (1) aerial surveys and (2) ground line transects

for (j in 1:2)

{

logit(xi[i,j,1]) <- mu[j]

Y[i,j,1] ~ dbin(xi[i,j,1],N.nest[i,1]) # Count data

}

# Observed/unobserved nest data for aerial surveys and ground line

# transects and other targeted surveys

for (k in 1:K)

{

logit(rhonest[i,k,1]) <- zeta

zi[i,k,1] <- O.nest[i,1]*rhonest[i,k,1]

Z.nest[i,k,1] ~ dbern(zi[i,k,1]) # Occurrence data

}

}

# SUBSEQUENT ABUNDANCE (TIME PERIOD 2 AND 3)

for (i in 1:I)

{

for (t in 2:T)

{

# LEVEL 1 (LATENT ORANGUTAN POPULATION)

# Colonization rate

nbr[i,1,t-1] <- 0

for (j in 1:TOTNEIGH[i])

{

nbr[i,j+1,t-1] <- nbr[i,j,t-1]+N.ou[NEIGHBOUR[i,j],t-1]

}

neigh[i,t-1] <- nbr[i,(TOTNEIGH[i]+1),t-1]/TOTNEIGH[i]

log(delta[i,t-1]) <- chi+log(neigh[i,t-1])

R[i,t] ~ dpois(delta[i,t-1])

# Survival rate

logit(theta[i,t]) <- eta[1]+eta[2]*ALT[i]+eta[3]*pow(ALT[i],2) +eta[4]*DRY[i]+eta[5]*pow(DRY[i],2)+eta[6]*WET[i] +eta[7]*pow(WET[i],2)+eta[8]*DPA[i,t]+eta[9]*MS[i]

+eta[10]*FR[i,t]+eta[11]*(FR[i,t]*CFA[i,t])

S[i,t] ~ dbin(theta[i,t], N[i,t-1])

lambda[i,t] <- S[i,t] + R[i,t]

# Occupancy

logit(phi[i,t]) <- beta[1]+beta[2]*ALT[i]+ beta[3]*pow(ALT[i],2) +beta[4]*DRY[i]+beta[5]*pow(DRY[i],2)+beta[6]*WET[i] +beta[7]*pow(WET[i],2)+beta[8]*DPA[i,t]+beta[9]*MS[i]

+beta[10]*FR[i,t]+beta[11]*(FR[i,t]*CFA[i,t])

O[i,t] ~ dbern(phi[i,t])

# Latent abundance

Nu[i,t] <- lambda[i,t]*O[i,t]

N.ou[i,t] ~ dpois(Nu[i,t])

# LEVEL 2 (ORANGUTAN OBSERVATION)

# Observed/unobserved orangutan data from interview survey

for (m in 1:M)

{

logit(rhoou[i,m,t]) <- upsilon[1] + upsilon[2]*FE[i,m]

zi[i,m,t] <- rhoou[i,m,t]*O[i,t]

Z.ou[i,m,t] ~ dbern(zi[i,m,t]) # Occurrence data

}

# LEVEL 3 (LATENT ORANGUTAN NEST POPULATION)

# Abundance and nest relationship

psi[i,t] <- gamma0+gamma1[1]*MGV[i,t]+gamma1[2]*PT[i,t]+ gamma1[3]*LOWL[i,t]+gamma1[4]*MONT[i,t]+gamma1[5]*FRGM[i,t]

N.nest[i,t] ~ dpois(N.ou[i,t]*psi[i,t]*100)

O.nest[i,t] <- step(N.nest[i,t]-1)

# LEVEL 4 (NEST OBSERVATION)

# Count data for (1) aerial surveys and (2) ground line transects

for (j in 1:2)

{

logit(xi[i,j,t]) <- mu[j]

Y[i,j,t] ~ dbin(xi[i,j,t],N.nest[i,t]) # Count data

}

# Observed/unobserved nest data for aerial surveys and ground line

# transects and other targeted surveys

for (k in 1:K)

{

logit(rhonest[i,k,t]) <- zeta

zi[i,k,t] <- O.nest[i,t]*rhonest[i,k,t]

Z.nest[i,k,t] ~ dbern(zi[i,k,t]) # Occurrence data

}

}

}

}"

# INPUT

# ----------------------------------------------------------------------------

# PARAMETERS:

# I = total number of grid cell (1 km resolution)

# T = total time period (3)

# K = total number of sub-cell within grid cell (K=25 for subgrid size of

# 200 m)

# M = total number of respondent in the interview survey (M=10)

# NEIGHBOUR[i,j] = neighbor indices for grid cell i

# TOTNEIGH[i] = total number of neighbors for grid cell i

# ----------------------------------------------------------------------------

# DATA:

# Y[i,j,t] = count data for grid cell i, time period t, and survey type j

# (aerial surveys and ground line transects)

# Z.nest[i,k,t] = nest occurrence data from aerial and ground line

# transects and other targeted surveys for sub-cell k, grid cell i,

# and time period t

# Z.ou[i,m,t] = orangutan occurrence data from interview surveys for

# respondent m at grid cell i and time period t

# ----------------------------------------------------------------------------

# PREDICTORS

# STATIC PREDICTORS:

# ALT[i] = altitude at grid cell i

# DRY[i] = long-term mean monthly rainfall during the dry season (May to

# September) at grid cell i

# WET[i] = long-term mean monthly rainfall during the dry season (November

# to March) at grid cell i

# MS[i] = proportions of Muslim populations at grid cell i

# FE[i,m] = the frequency of respondent m for entering the forest around

# the village at grid cell i (1 for more than once a month and 0 for

# less than once a month).

# DYNAMIC PREDICTORS:

# FR[i,t] = forest cover at grid cell i and time period t

# DPA[i,t] = mean nearest distance to protected areas for grid cell i and

# time period t

# CFA[i,t] = mean nearest distance to newly converted forest to industrial

# agriculture for grid cell i and time period t

# MGV[i,t] = location on mangrove forest (binary) at grid cell i and time

# period t

# PT[i,t] = location on peat forest (binary) at grid cell i and time

# period t

# LOWL[i,t] = location on lowland forest (binary) at grid cell i and time

# period t

# MONT[i,t] = location on mantane forest (binary) at grid cell i and time

# period t

# FRGM[i,t] = location on fragmented forest (binary) at grid cell i and

# time period t

# ----------------------------------------------------------------------------

**Supplementary Method 3**

Land uses on Borneo

We appraised five land use categories on Borneo: 1) protected areas (PA), 2) logging concessions on natural forests (LOGG), 3) industrial timber plantation concessions (ITP), 4) oil palm concessions (OPP), and 5) outside protected areas and without concessions (OTHER). Protected areas (PA) in Indonesia include strict protected areas (national parks, nature reserves, wildlife reserves) under the Indonesian Ministry of Environment and Forestry and watershed protection forest (*Hutan Lindung*) under the District Forestry Agency ^1^. For Sabah, this category includes protection forest reserves under the Sabah Forestry Department (permanent forest reserves (class I), mangrove forest reserves (class V), virgin jungle reserves (class VI) and wildlife reserves (class VII)), wildlife sanctuaries under the Sabah Wildlife Department, and national parks under Sabah Parks ^2^. For Sarawak, this mainly includes protected forest under Sarawak Forestry Department (national parks, nature reserves and wildlife sanctuaries) ^3-10^. Logging concessions (LOGG) are forest areas allocated to companies possessing logging licenses to sustainably extract timber from natural forests ^1-8^. Industrial timber plantation concession (ITP) is a license granted by a government to develop a piece of land into an industrial monoculture tree plantation of fast-growing species, primarily for pulp, paper and rubber production (*Acacia mangium*, *Hevea*, or *Eucalyptus spp*.) ^1-8^. Oil palm concession (OPP) is a license granted by a government to allow the establishment of industrial monocultures of oil palm (*Elaeis guineensis*) ^1, 4-8, 11, 12^.

Description of PA, LOGG, ITP and OPP for Kalimantan, Sabah, and Sarawak, and the sources of these data are provided in Table M3. Maps of the distributions of PA, LOGG, ITP, and OPP on Borneo for three time periods between 1997 and 2015 are shown in Fig. M3 and are available at https://figshare.com/s/265c64b6d15702f38e0b ^13^.


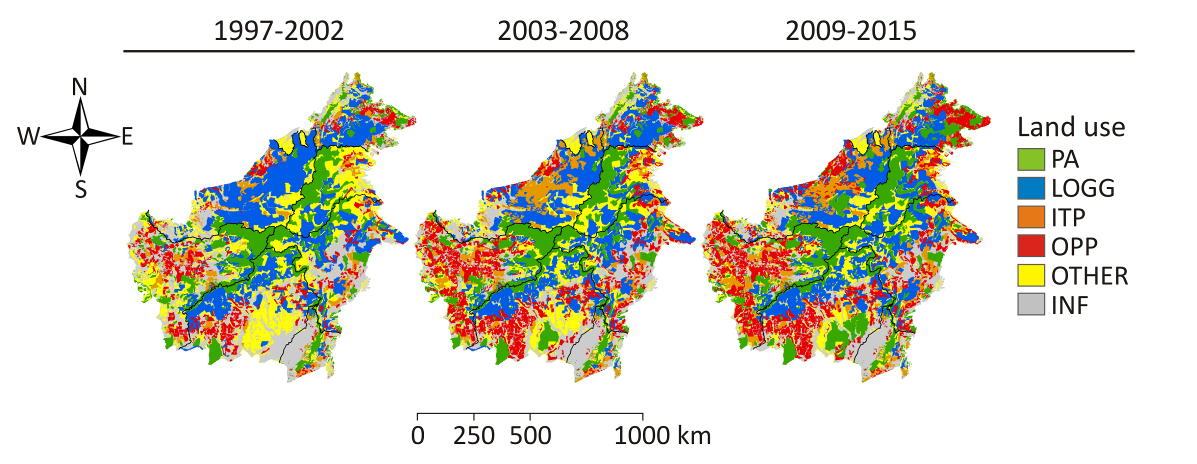


**Fig. M3.** Distributions of protected areas (PA), logging concessions on natural forests (LOGG), industrial timber plantation concessions (ITP), oil palm concessions (OPP), infrastructure, roads and urban areas (INF), and outside PA and INF and without concessions, mostly small-scale agriculture and smaller forest patches (OTHER) on Borneo for three time periods between 1997 and 2015. Black lines indicate the regional boundaries (i.e. national, state, and provincial boundaries). These maps are available at https://figshare.com/s/265c64b6d15702f38e0b ^13^.

**Table M3.** Description of protected areas (PA), logging concessions on natural forests (LOGG), industrial timber plantation concessions (ITP), oil palm concessions (OPP) for Kalimantan, Sabah and Sarawak.

| **Land use** | **Region** | **Description** | **Data sources** |
| --- | --- | --- | --- |
| Protected areas (PA) | *Kalimantan*  *(Indonesia)* | Includes strict protected areas (national parks, nature reserves, wildlife reserves) under the Indonesian Ministry of Forestry and watershed protected forest (*Hutan Lindung*) under the District Forestry Agency. | 1 |
|  | *Sabah*  *(Malaysia)* | Includes protection forest reserves under the Sabah Forestry Department (permanent forest reserves (class I), mangrove forest reserves (class V), virgin jungle reserves (class VI) and wildlife reserves (class VII)), wildlife sanctuaries under the Sabah Wildlife Department, and national parks under Sabah Parks | 2 |
|  | *Sarawak*  *(Malaysia)* | Includes protected forest under Sarawak Forestry Department (national parks, nature reserves and wildlife sanctuaries). | 3―10 |
| Logging concessions on natural forest (LOGG) | *Kalimantan*  *(Indonesia)* | Issued by the Ministry of Forestry under IUPHHK-HA license and the concessions are granted inside production forest. | 1 |
|  | *Sabah*  *(Malaysia)* | Granted inside commercial forest reserves designated for reduced-impact logging (class II) and the license is issued by the Sabah Forestry Department | 2―4, 6, 7 |
|  | *Sarawak*  *(Malaysia)* | Issued by the Sarawak Forestry Department and the concessions are allocated inside permanent forest estate. | 3―8 |
| Industrial timber plantation concessions (ITP) | *Kalimantan*  *(Indonesia)* | Issued by the Ministry of Forestry under IUPHHK-HT license and the concessions are granted inside production forest. | 1 |
|  | *Sabah*  *(Malaysia)* | Issued by the Sabah Forestry Department and over the last decades the concessions has been mainly operated by three timber plantation companies: Sabah Forest Industries, SAFODA (Sabah Forestry Development Authority) and Sabah Softwood. | 2―4, 6, 7 |
|  | *Sarawak*  *(Malaysia)* | Issued by the Sarawak Forestry Department under License for Planted Forest (LPF). | 4―8 |

**Table M3.** (cont.)

| **Land use** | **Region** | **Description** | **Data sources** |
| --- | --- | --- | --- |
| Oil palm concessions (OPP) | *Kalimantan*  *(Indonesia)* | Issued by the Department of Estate Crops within the Ministry of Agriculture under Izin Usaha Perkebunan (IUP) or Hak Guna Usaha (HGU) license. | 1, 11, 12 |
|  | *Sabah*  *(Malaysia)* | Issued by the Environmental Conservation Department and has to pass a series of Environmental Impact Assessment study. | 6 |
|  | *Sarawak*  *(Malaysia)* | Issued earlier by the state government solely under Provisional Lease (PL) license, but now oil palm estate can also be established in area with License for Planted Forest (LPF) inside permanent forest estate. | 4―8 |
| OTHER | *Kalimantan*  *(Indonesia)* | Forest outside PA, concessions (LOGG, ITP and OPP), and infrastructure, roads and urban areas (*Areal Penggunaan Lain* (APL)) | 1 |
|  | *Sabah (Malaysia)* | Permanent forest estate outside PA and concessions (LOGG, ITP and OPP) | 2 |
|  | *Sarawak (Malaysia)* | Permanent forest estate outside PA and concessions (LOGG, ITP and OPP) | 3―8 |

**References for Supplementary Method 3**

1. Ministry of Forestry. *Data dan Informasi Pemanfaatan Hutan* (Forest Planology Agency, Ministry of Forestry, Jakarta, Indonesia, 1998, 2000, 2003, 2007, 2010, 2012). Available at http://www.dephut.go.id (accessed 21 August 2015).
2. Sabah Forestry Department. *Sabah Forestry Department Annual Report* (Sabah, Malaysia, 2001, 2006, 2013). Available at http://www.forest.sabah.gov.my/publication/annual-reports (accessed 10 October 2015).
3. Hardiono, M. & Alfred, R.J. *Borneo: Treasure Island at Risk* (WWF Germany, 2005).
4. Raman, M., van Schaik, A., Richter, K. & de Clerck, P. *Malaysian Palm Oil - Green Gold or Green Wash?* (Friends of the Earth England, Wales and Northern Ireland, United Kingdom, 2008). Available at https://milieudefensie.nl/publicaties/rapporten/malaysian-palm-oil-green-gold-or-green-wash-summary.
5. Faeh, D. *Development of Global Timber Tycoons in Sarawak, East Malaysia - History and Company Profiles* (Bruno Manser Funds, Switzerland, 2011). Available at http://www.bmf.ch/upload/berichte/bmf_report_sarawak_timber_tycoons_1.pdf.
6. Hamzah, K.A., Malik, R.A. & Joseph, K.T. *Malaysia Land-use Change in the Oil Palm Sector* (The 9th Annual Roundtable Meeting on Sustainable Palm Oil, Sabah, Malaysia, 2011).
7. Wyn, L.T. *Malaysia Illegalities in Forest Clearance for Large-scale Commercial Plantations* (The Family of Forest Trends Initiatives, United Kingdom, 2012). Available at http://www.forest-trends.org/documents/files/doc_4195.pdf.
8. Cramb, R. *A Malaysian Land Grab? The Political Economy of Large-scale Oil Palm Development in Sarawak* (The Land Deal Politics Initiative, Netherland, 2013). Available at http://www.iss.nl/fileadmin/ASSETS/iss/Research_and_projects/Research_networks/LDPI/LDPI_WP_50.pdf.
9. Global Forest Watch (GFW). *Sarawak Protected Areas 2016*. Available at http://gis-gfw.wri.org/arcgis/rest/services/country_data/mys/MapServer (accessed 18 January 2016).
10. Sarawak Forestry Corporation. *Sarawak National Parks and Reserves* (Kuching, Sarawak, Malaysia, 2016). Available at http://www.sarawakforestry.com/htm/snp-np.html (accessed 2 February 2016).
11. Ministry of Agriculture. *Daftar Perusahaan Perkebunan Penerima Izin Usaha Perkebunan (IUP-B, IUP-P dan IUP)* Department of Estate Crops, Ministry of Agriculture, Jakarta, Indonesia, 2011). Available at http://ditjenbun.pertanian.go.id/pascapanen (accessed 10 January 2016).
12. World Resource Institute. *Indonesia Oil Palm Concessions* (2014) Available at http://data.globalforestwatch.org/datasets/f82b539b9b2f495e853670ddc3f0ce68_2 (accessed 21 January 2016).
13. Santika, T. *Land Uses on Borneo*. figshare (2016). Available at https://figshare.com/s/265c64b6d15702f38e0b.

**Supplementary Data 1**

Funding acknowledgements

TS thanks The Nature Conservancy (TNC) Indonesia and Arcus Foundation for funding support.

KAW thanks the Australian Research Council Centre of Excellence and Future Fellowship programs for funding support.

GLB thanks The University of Aberdeen (Expedition, Alumni Annual and Small Grants Funds), the Royal Geographical Society (with Institute of British Geographers), the Royal Scottish Geographical Society, the Gilchrist Educational Trust, the Orangutan Foundation UK and the North of England Zoological Society at Chester Zoo for funding support.

AJM thanks the Louis Leakey Foundation, Orangutan Conservancy, Hellman Foundation, Mohamed bin Zayed Foundation, AZA Ape TAG, Seneca Park Zoo, University of California, Davis, Victoria University of Wellington, and University of Michigan for funding support, Universitas Tanjungpura for counterpart support, and the many students, researchers, and field assistants who worked at the Cabang Panti Research Station over the past two decades.

AER thanks the NSERC (Natural Sciences and Engineering Research Council; in Canada), the Leakey Foundation, York University (Toronto), and Indianapolis Zoo for funding support.
